# Transcriptional Profiling of Bone Marrow Stromal Cells in Response to *Porphyromonas gingivalis* Secreted Products

**DOI:** 10.1371/journal.pone.0043899

**Published:** 2012-08-24

**Authors:** Durga Reddi, Georgios N. Belibasakis

**Affiliations:** 1 Centre for Adult Oral Health, Barts and the London Institute of Dentistry, Queen Mary University of London, London, United Kingdom; 2 Oral Microbiology and Immunology, Institute of Oral Biology, Center of Dental Medicine, University of Zürich, Zürich, Switzerland; University of Toronto, Canada

## Abstract

Periodontitis is an infectious inflammatory disease that destroys the tooth-supporting (periodontal) tissues. *Porphyromonas gingivalis* is an oral pathogen highly implicated in the pathogenesis of this disease. It can exert its effects to a number of cells, including osteogenic bone marrow stromal cells which are important for homeostastic capacity of the tissues. By employing gene microarray technology, this study aimed to describe the overall transcriptional events (>2-fold regulation) elicited by *P. gingivalis* secreted products in bone marrow stromal cells, and to dissect further the categories of genes involved in bone metabolism, inflammatory and immune responses. After 6 h of challenge with *P. gingivalis*, 271 genes were up-regulated whereas 209 genes were down-regulated, whereas after 24 h, these numbers were 259 and 109, respectively. The early (6 h) response was characterised by regulation of genes associated with inhibition of cell cycle, induction of apoptosis and loss of structural integrity, whereas the late (24 h) response was characterised by induction of chemokines, cytokines and their associated intracellular pathways (such as NF-κB), mediators of connective tissue and bone destruction, and suppression of regulators of osteogenic differentiation. The most strongly up-regulated genes were lipocalin 2 (LCN2) and serum amyloid A3 (SAA3), both encoding for proteins of the acute phase inflammatory response. Collectively, these transcriptional changes elicited by *P. gingivalis* denote that the fundamental cellular functions are hindered, and that the cells acquire a phenotype commensurate with propagated innate immune response and inflammatory-mediated tissue destruction. In conclusion, the global transcriptional profile of bone marrow stromal cells in response to *P. gingivalis* is marked by deregulated homeostatic functions, with implications in the pathogenesis of periodontitis.

## Introduction

Periodontitis is as a condition characterised by a bacterially-induced inflammatory destruction of the tooth-supporting (periodontal) tissues, including the alveolar bone and the interconnecting periodontal ligament. *Porphyromonas gingivalis* is a Gram-negative anaerobe highly associated with chronic periodontitis. It is frequently and at high levels detected in diseased sites, particularly with active disease progression, but rarely or at low levels in healthy sites [1], [2]. *P. gingivalis* is considered notorious for its capacity to manipulate the host innate immune and inflammatory responses, as a strategy to survive and prevail into the periodontal tissue habitat [3]–[5]. A number of virulence factors, such as its cysteine proteinases (gingipains), lipopolysaccharide (LPS) and fimbriae [6], collectively contribute to these properties of *P. gingivalis*.

Bone marrow stromal cells have osteogenic protential and can give rise to bone forming osteoblasts under the appropriate stimulation [7]. Among other cells types, bone marrow stromal cells are also a target for *P. gingivalis*. A number of studies have focused on the capacity of *P. gingivalis* to trigger mechanisms of bone resorption in these cells [8]–[10], which is a key histopathological trait of periodontitis. There are also studies addressing the global effect of *P. gingivalis* on stromal cells, using gene microarray technology. These have demonstrated that approximately 360 genes were up-regulated (greater than 2-fold) in response to *P. gingivalis*, including genes encoding for chemokines, pro-inflammatory cytokines and matrix metalloproteinases (MMPs) [11]. Moreover, they have identified signalling pathways that may be involved in the pro-inflammatory responses to *P. gingivals*, predominantly the activation of transcription factors nuclear factor-kappaB (NF-κB) and activator protein (AP)-1 [12].

Nevertheless, the full range of transcriptional changes inflicted in bone marrow stromal cells by *P. gingivalis* with potential implications in the pathogenesis of periodontal disease is yet to be determined. Therefore, by employing gene microarray technology, the aim of the present study was to describe the overall transcriptional effects of *P. gingivalis* on osteogenic bone marrow stromal cells over time, dissecting the categories of genes involved in bone metabolism, inflammatory and immune responses, which are all associated with the pathogenesis of periodontal disease.

## Materials and Methods

### Preparation and Growth of *Porphyromonas gingivalis*

*Porphyromonas gingivalis* W50 strain (provided by Centre of Immunology and Infectious Disease, Blizard Institute of Cellular and Molecular Science, Barts and the London School of Medicine and Dentistry) was grown on blood agar plates supplemented with 5% horse blood in an anaerobic environment containing 80% nitrogen, 10% hydrogen and 10% carbon dioxide at 37°C for 3 days, before then being sub-cultured into 10 ml of media consisting of brain heart infusion (BHI) broth supplemented with 5 µg/ml of hemin. The following day, the 10 ml culture was inoculated into 90 ml of fresh media. This was considered day 0 of the culture. At day 6, the bacterial cultures were centrifuged at 8500 rpm for 45 min at 4°C, and the resulting bacterial supernatant was removed, aliquoted and stored at −80°C for further use to challenge the cells. In further mentions within the text, this preparation is referred to simply as “*P. gingivalis*", for reasons of clarity.

### Bone Marrow Stromal Cell Culture and Challenge with *Porphyromonas gingivalis*

An established murine bone marrow stromal cell line, W20-17 [7], was used in this experimental system, as previously described [9], [10]. Cell culture was carried out in alpha minimum essential medium (α-MEM, Gibco-BRL) supplemented with 10% v/v foetal calf serum, 50 U/ml penicillin, 50 µg/ml streptomycin (Gibco-BRL) and 50 µg/ml of ascorbic acid (Sigma). The cell cultures were incubated at 37°C in an atmosphere of 5% CO_2._ For sub-culturing, the cells were detached from the bottom of the culture flask with 0.1% Trypsin/EDTA (Gibco) and centrifuged at 1000 rpm for 5 min at room temperature. For the experiments, the cells were seeded at a density of 2×10^4^ cells/cm^2^ and left to attach for at least 18 h before being challenged with the *P. gingivalis* culture supernatants. When thawed, the aliquoted bacterial supernatants were filter-sterilized (0.2 µm filter) and added into the cell culture at 5 µg/ml total bacterial protein concentration. The cultures were further incubated at 37°C in the presence of 5% CO_2_ for 6 h and 24 h. The selection of this concentration of *P. gingivalis* and challenge period was based on earlier studies demonstrating effects on gene expression by the cells, in the absence of cytotoxicity [9], [10]. Untreated cell cultures served as controls. For each group, four biological replicates were used.

### Gene Microarray Protocol

To investigate the gene expression profile of W20-17 bone marrow stromal cells challenged with *P. gingivalis*, the Illumina Whole-Genome Expression Assay was used (MouseRef-8 v2.0 Expression BeadChips). The Illumina technology consists of oligonucleotides immobilised to beads in microwells contained in the array that bind to complimentary sequences contained in the target template. After the cells were challenged with 5 µg/ml protein concentration of *P. gingivalis* supernatant for 6 h and 24 h, the cell culture supernatant was removed and total RNA was then extracted and collected from the cells by the RNeasy Mini Kit (QIAGEN). This was performed for all four biological replicates in each group. From each sample, 250 ng of high quality total RNA were biotin-labelled with a single round amplification using the Illumina total preparation kit (LifeTech). Spike RNA controls were also included within samples prior to amplification, in order to confirm a successful reaction. The biotin-labelled cRNA was then quantified using a Nanodrop spectrometer. To confirm the quality of the RNA, the fragment size distribution of ribosomal RNA was assessed by electrophoresis on a RNA nano-chip on the Bioanalyser (Agilent). A total of 750 ng of cRNA was then hybridised to the MouseRef-8 v2 array (Illumina) according to the manufacturer’s instructions and data was visualised on the Bead Array Reader (Illumina). This assay was performed at the core facilities of the Genome Centre of Barts and The London School of Medicine and Dentistry.

### Gene Microarray Data Analysis

Intensity data from the Bead Array Reader was imported into the BeadStudio software (Illumina) to be analysed. Quality control checks were performed based on spiked-in controls. Data fulfilling the quality criteria were then quantile-normalised before grouping replicates and performing group differential analysis using the Beadstudio software. Further to this, a Bonferonni statistical test was performed to adjust for a large number of p values, which were then converted to differential (diff) scores. This takes into account both the p value and the difference between the average signal of the reference and comparison group (in this case the control group versus the *P. gingivalis*-challenged group). A p value of 0.001 is equivalent to a diff score of ±33. For further stringency, an arbitrary cut-off diff score of ±65 was chosen. Over 24,000 transcripts were assayed and to narrow the frame of reference a cut-off threshold of 2-fold regulation (up- or down-) was chosen. Where applicable, a small number of genes of interest lower than 2-fold were also included. The list of genes together with fold changes was uploaded into the Ingenuity software (Ingenuity Systems®, http://www.ingenuity.com), which was then used to sort the list of genes into broad cellular categories.

### Validation using Quantitative TaqMan® Real-time PCR (qPCR)

To validate the microarray results, the mRNA expression levels of a selected number of genes were alternatively quantified by qPCR, on cDNA prepared from the samples. For the amplification reactions, Applied Biosystems (ABI) TaqMan® Gene Expression Assays and the ROX mastermix were used. The qPCR reactions and analyses were performed in an ABI Prism 7900HT Sequence Detection System. The amplification conditions were 10 min at 95°C, followed by 40 cycles at 95°C for 15 sec and 60°C for 1 min. The TaqMan® Gene Expression Assays IDs for the studied target genes were as follows: TNFSF11 (RANKL): Mm00441908_m1, TNFRSF11B (OPG): Mm00435452_m1, COX-2: Mm00478377_g1, SAA3: Mm00441203_m1, and LCN2: Mm01324470_m1. To be able to normalise the obtained target gene Ct values, a housekeeping gene screen was performed in cDNA from W20-17 cells, using a series of 12 murine housekeeping gene candidates. The screen performed indicated that the eukaryotic translation initiation factor 4A, isoform 2 (EIF4A2: Mm008343457_g1) and cytochrome 1 (CYC1: Mm00470540_g1) gene expressions were the most stable and consistent among all, and were used to normalise the data. For each sample, the relative target gene expression was determined as the ΔCt value, which is the difference between the target gene Ct and the average Ct of the selected two housekeeping genes.

### Statistical Analysis

The data on qPCR validation experimentations were analysed by Student’s t test in order to determine statistical significance of differences between the corresponding *P. gingivalis* challenged and control groups.

## Results and Discussion

The global transcriptional effects of *P. gingivalis* on bone marrow stromal cells were evaluated by an Illumina bead gene microarray (MouseRef-8 v2.0 Expression BeadChips). W20-17 bone marrow cells were challenged with 5 µg/ml of *P. gingivalis* W50 culture supernatant, over a 6 h or 24 h time period. It is anticipated that the selection of these two time-points correspond respectively to periods of early and late responses to *P. gingivalis*. Moreover, it is know from earlier studies in the present experimental system that *P. gingivalis* challenge does not elicit significant cytotoxicity during these periods [9], [10].

Relative changes of gene expression induced by *P. gingivalis* in bone marrow stromal cells were determined by normalising against the corresponding control group. More than 24,000 transcripts were assayed. To narrow-down the frame of reference, a cut-off threshold of 2-fold was selected. A small number of genes exhibiting lower than 2-fold regulation were also included in the analyses, since these were of focus in earlier gene expression studies employing the present experimental model [9], [10], in order to validate these earlier results.

### Global Gene Regulation

The collective data indicates that 480 genes were regulated more than 2-fold after 6 h (Table S1), whereas 364 genes were regulated more than 2-fold after 24 h (Table S2). In particular, after 6 h of challenge, *P. gingivalis* caused up-regulation of 271 genes and down-regulation of 209 genes, whereas after 24 h of challenge, 259 genes were up-regulated and 109 genes were down-regulated.

### Functional Classification of Regulated Genes

The genes that were regulated more than 2-fold were further categorised according to their known cellular function using the Ingenuity Pathway Analysis (Ingenuity Systems®, http://www.ingenuity.com). The top five cellular functions and the corresponding percentage of their up- and down-regulated genes at both time-points are displayed in Table 1. Interestingly, a bi-phasic response was demonstrated by this data. An early 6 h-response to *P. gingivalis* was characterised by regulation of genes associated with cell movement, growth, proliferation and intracellular signalling. However, the late 24 h time-point was characterised by the regulation of genes associated with the immune and inflammatory response, including the categories of immunological disease, antigen presentation, cell-mediated and humoral immunity. A marked overlap between the two time-points was the regulation of genes with associated functions in cancer. This should not imply that *P. gingivalis* is involved in carcinogenesis, as this category is broad enough to include genes with roles in cell growth, proliferation and apoptosis, functions which are all affected by *P. gingivalis* challenge. Further focus is placed on the gene categories with ascribed roles in inflammatory processes and skeletal/muscle disorders (i.e. bone metabolism), according to Table 2. The reason for this filtering is that periodontal disease has combined inflammatory and bone destruction traits. Therefore, looking in more detail into these categories is of relevance to the disease. Within these categories, results are presented and discussed according to clustering of the regulated genes based on cellular and molecular function.

**Table 1:** Top five regulated gene categories according to cellular function.

Genes regulated after 6 h				
Cellular function	Up-regulated	Down-regulated		
Cancer	33%	22%		
Cellular Movement	16%	10%		
Cellular growth and proliferation	24%	20%		
Cell-to-cell signalling	14%	9%		
Haematological system (development and function)	17%	6%		
**Genes regulated after 24 h**				
**Cellular function**	**Up-regulated**		**Down-regulated**	
Cancer	36%		33%	
Immunological disease	25%		9%	
Antigen presentation	29%		7%	
Cell-mediated immune response	28%		10%	
Humoral immune response	26%		7%	

The percentage of genes regulated within each category is provided.

**Table 2:** Genes regulated by *P. gingivalis* and associated with the categories of Skeletal/Muscle disorders and inflammatory response.

Gene category	Fold Change (6 h)	Fold Change (24 h)	Skeletal & Muscle Development & Function	Skeletal & Muscle Disorders	Inflammatory Response
**Apoptosis genes**					
Caspase 4 (CASP4)	2.8	4.3			X
Tumor necrosis factor receptor superfamily member 6 (FAS)	7.8	11.5		X	X
**Cell cycle genes**					
Cyclin D1		**−3.1**			X
p16	2.0		X	X	
p21	5.3		X	X	X
**Chemokines and cytokines**					
CCL17	8.0	3.1		X	X
CCL2	17.6	20.7	X	X	X
CCL3	3.3	40.0	X	X	X
CCL5	9.0	86.8		X	X
CCL7	2.8	3.7	X	X	X
CCL9	7.2	8.3	X	X	
CX3CL1	10.6	4.7			X
CXCL1	23.1	29.2		X	X
CXCL10	7.1	5.9		X	X
CXCL15		6.8		X	X
CXCL2	2.6	3.0		X	X
CXCL4		2.3			X
CXCL5		112.0			X
CXCL7		16.0			X
CXCL9	10.5	35.0		X	X
Interleukin 1, alpha (IL-1α)		6.5	X	X	X
Interleukin 15 (IL-15)		−**2.3**		X	X
Interleukin 6 (IL-6)		23.2	X	X	X
Macrophage colony stimulating factor 1 (M-CSF1)	2.7		X	X	X
Macrophage colony stimulating factor 2 (M-CSF2)	2.4		X	X	X
Receptor activator of nuclear factor-κB ligand (RANKL)		1.9	X	X	X
Tumor necrosis factor (ligand) superfamily, member 13b (TNFSF13B)		3.4		X	X
**Extracellular matrix genes**					
Matrix GLA protein (MGP)		**−2.9**	X		
**Growth factor genes**					
Bone morphogenic protein 4 (BMP4)	**−2.2**		X	X	
Hepacyte growth factor (HGF)		2.3		X	X
Vascular endothelial growth factor (VEGF)	2.1	2.8	X	X	X
**Guanosine triphosphate enzymes (GTPase) genes**					
GTP cyclohydrolase 1 (GCH1)	9.0			X	
Guanylate binding protein 2 (GBP2)	4.4	16.3			X
**Hormones**					
**Inflammation genes**					
Cyclooxygenase-2 (COX-2)	3.2	2.3	X	X	X
Haptoglobin (Hp)	2.6	7.5			X
Hemopexin (Hpx)		7.0			X
Lipocalin 2 (Lcn2)	33.6	354.8			X
Lipolysaccharide binding protein (LBP)		2.4			X
Myeloid differentiation protein-2 (MD-2)	−2.3				X
Prostaglandin E synthase (PTGES)	3.2				X
Serum amyloid A3 (SAA3)	144.8	143.9		X	X
Superoxide dismutase 2 (SOD2)	2.3	3.2	X	X	X
**Immune related genes**					
Cathelicidin antimicrobial peptide (CAMP)		9.4			X
Complement component 1s (C1s)		2.6		X	X
Complement component 3 (C3)		14.3		X	X
Complement component 4B (C4B)		2.3		X	X
Complement factor B (CFB)		19.9		X	X
DHX58		2.7			X
Interferon gamma inducible protein 47 (IFI47)	3.1	2.9			X
Interferon induced transmembrane protein 3 (IFITM3)		3.2			X
Interferon, alpha-inducible protein 27 (IFI27)		2.6			X
Macrophage antigen CD68		2.0			X
Major histocompatibility 2, M region locus 3 (H2–M3)	2.0	2.0			
Major histocompatibility complex, class I, C (HLA-C)		2.6		X	X
Major histocompatibility complex, class I, E (HLA-E)		2.8		X	
Major histocompatibility complex, class II, DM alpha (HLA-DMA)	2.0			X	X
Major histocompatibility complex, class II, DQ beta 2 (HLA-DQβ2)		3.4		X	X
Pentraxin 3, long (PTX3)	11.9	32.0			X
Retinoic acid-inducible gene 1 protein (RIG-1)		2.2			X
S100 calcium binding protein A8 (S100A8)		3.4		X	X
Spondin 2 (SPON2)*	2.2	2.8			
Ubiquitin D (UBD)*	2.2	3.2			
**Proteases and related genes**					
A disintegrin and metalloproteinase domain 12 (ADAM12)	**−2.4**				X
A disintegrin-like and metalloproteinase with thrombospondin (ADAMTS7)		2.1		X	
Cathepsin L (CTSL)	2.2		X		X
Matrix metalloproteinase 12 (MMP12)		2.9			X
Matrix metalloproteinase 13 (MMP13)	9.3	8.4		X	X
Matrix metalloproteinase 13 (MMP17)	3.4			X	X
Matrix metalloproteinase 14 (MMP14)	-2.2			X	X
Matrix metalloproteinase 3 (MMP3)		35.8		X	X
Matrix metalloproteinase 9 (MMP9)		3.0		X	X
Proteasome subunit, beta type, 8 (PSMB8)	2.1				X
Secretory leukocyte peptidase inhibitor (SLPI)	4.9	19.5			X
Tissue inhibitor of metalloproteinase 1 (TIMP1)	2.1			X	
**Receptor genes**					
CD44	2.2		X	X	X
Cluster of differentiation 14 (CD14)		2.1			X
Interferon Receptor 2 (IFNAR2)		2.7		X	X
Interleukin 1 receptor antagonist (IL1RN)	**−3.9**		X	X	X
Interleukin 10 receptor, beta (IL10RB)		2.0			X
Interleukin 13 receptor, alpha 1 (IL-13RA1)		4.1			X
Interleukin 13 receptor, alpha 2 (IL-13RA2)		11.0			X
Osteoprotegerin (OPG)	−4.0		X	X	
Toll like receptor 2 (TLR-2)	3.7	12.6	X	X	X
Tumor necrosis factor receptor superfamily, member 9 (TNFRSF9)		3.9		X	X
**Structural genes**					
Caveolin 1 (CAV1)		**−2.1**		X	X
Vimentin (VIM)		**−2.1**		X	
**Signal transduction genes**					
B-cell lymphoma 3-encoded protein (BCL3)		2.7			X
Growth arrest and DNA-damage-inducible, beta (GADD45B)		2.6		X	
Immediate early response 3 (IER3)	4.9	5.5			X
Inhibitor of kappa light polypeptide gene enhancer in B-cells, kinase epsilon (IκBKE)		1.7			X
Inhibitor of kappa light polypeptide gene enhancer in B-cells, kinase gamma (IκBKG)		1.7			X
Interleukin-1 receptor-associated kinase 3 (IRAK3)		2.3		X	X
Janus kinase 2 (JAK2)		6.9			X
Myeloid differentiation primary response gene (88) (MYD88)		1.7	X	X	X
Nuclear factor of kappa light polypeptide gene enhancer in B-cells inhibitor (NF-κBIA)	4.6	5.7	X	X	X
Nuclear factor of kappa light polypeptide gene enhancer in B-cells inhibitor (NF-κBIZ)	11.2	9.6			X
Receptor-interacting serine-threonine kinase 2 (RIPK2)	2.4	2.9			X
Suppressor of cytokine signalling 3 (SOCS3)	2.3	4.5	X	X	X
TNFAIP3 interacting protein 1 (TNIP1)	2.7	5.9			X
Tumor necrosis factor, alpha-induced protein 3 (TNFAIP3)	2.4	4.8	X	X	X
WNT1 inducible signalling pathway protein 2 (WISP2)	**−2.6**		X		
V-rel reticuloendotheliosis viral oncogene homolog B (REL-B)		4.0			X
**Transcription factor and inhibitor genes**					
CCAAT/enhancer-binding protein beta (CEBPβ)	3.4	4.2	X	X	X
FBJ murine osteosarcoma viral oncogene homolog (c-Fos)	1.7	1.4	X	X	X
Hairy-related transcription factor 1 (HEY1)	2.1				X
Jun B proto-oncogene (JUNB)	2.6	5.4	X		X
Nuclear factor of kappa light polypeptide gene enhancer in B-cells 1 (NF-κB1)		2.6	X		X
Nuclear factor of kappa light polypeptide gene enhancer in B-cells 2 (NF-κB2)		2.2			X
Sex determining region Y box 9 (SOX-9)		**−3.1**	X	X	X
Signal transducer and activator of transcription 3 (STAT3)	2.6	2.6	X	X	X
V-rel reticuloendotheliosis viral oncogene homolog A (avian) (REL-A)		1.5			X

The genes regulated more than 2-fold by *P. gingivalis* were categorized according to ascribed roles in a) skeletal and muscle development and function, b) skeletal and muscle disorders, and c) inflammatory responses (horizontal categories). Within each of these thee categories, genes were sub-categorized according to ascribed cellular function (vertical categories). The fold-changes in expression (compared to control) at 6 h and 24 h are provided. The minus values (also in bold letters) indicate down-regulation. When a –fold change value is missing in one of the two time-points, the regulation of gene expression at that time-point was less that 2-fold.

### Apoptosis, Regulation of Cell Cycle and Structural Integrity

In response to *P. gingivalis* challenge, the transcription of three genes with involvement in apoptotic processes was regulated. These were namely caspase 4 (CASP4), tumor necrosis factor receptor superfamily member 6 (FAS) and engulfment adaptor PTB domain containing 1 (GULP1). These were all up-regulated at both 6 h and 24 h (Table 2, apoptosis related genes), suggesting mechanisms by which *P. gingivalis* challenge may induce the apoptosis. *P. gingivalis* has been previously shown to induce apoptotic cell death in fibroblasts, endothelial cells and epithelial cells [13]–[15], and that its gingipains in particular may have a role in this effect [15].

The expression of a number of genes that encode for cell cycle-regulatory proteins was also affected by *P. gingivalis*. After 6 h of challenge, the negative regulators of cell cycle p16 and p21 were up-regulated by 2.0- and 5.3-fold, respectively, while p18, another inhibitor of the cell cycle, was down-regulated by 2.0-fold. Cyclin D1, the only positive regulator of the cell cycle to be affected, was down-regulated by 3.1-fold over a 24 h period of challenge (Table 2, Cell cycle genes). Cyclin D1 is crucial for entry into the G1 phase, whereas p16 and p21 are responsible for arresting the cell cycle in G1 phase. Collectively, the effects on *P. gingivalis* on these genes would imply a possible G1 phase arrest of the cells. To this extent, there is evidence that *P. gingivalis* decreases cyclin D1 expression, causing G_1_ phase arrest of the cell cycle [16], [17]. This is shown to be concomitant to an up-regulation of p16 and p21 levels [17], and mainly attributed to the gingipains of *P. gingivalis* [16].

The expression of claudin 15 (CLDN15), a structural protein of tight junctions, was down-regulated by 2.9 and 3.2 at 6 h and 24 h, respectively. Other structural genes that were down-regulated at 24 h include caveolin 1 (CAV1), an integral membrane scaffolding protein, and vimentin (VIM), an intermediate filament (Table 2, Structural genes). Interestingly, the extracellular matrix GLA protein (MGP) normally associated with bone and cartilage was down-regulated by −2.9 fold at 24 h (Table 2, Extracellular matrix proteins).

Collectively, the results in the present experimental system indicate an overall positive effect of *P. gingivalis* on apoptosis, accompanied by a negative effect on cell cycle progression and cell-structural integrity, which are initiated at 6 h and culminated at 24 h. All of these events corroborate an involvement of *P. gingivalis* in the pathogenesis of periodontal disease, by early disruption of tissue homeostasis.

### Chemokine, Cytokines and other Inflammatory Mediators

The regulation of inflammatory chemokines and cytokines groups by *P. gingivalis* challenge was further investigated. Chemokines are essentially cytokines that are involved in recruiting leukocytes to inflammatory sites. Their recruitment is an act of protection against bacterial invasion, but over-stimulation can conversely lead to tissue destruction. In the present experimental system, after 24 h of challenge, the most strongly up-regulated chemokine was LPS-induced CXC chemokine (also named CXCL5) by a marked 112.0-fold, followed by chemokine C-C motif ligand 5 (CCL5) by 86.8-fold, and chemokine C-X-C motif ligand 9 (CXCL9) by 35.0-fold (Table 2, Chemokine and cytokines). Other up-regulated chemokines include CCL17, CCL2, CCL3, CCL7, CCL9, CXCL1, CXCL10 and CXCL2. These findings are in line with earlier studies on bone marrow stromal cells, whereby *P. gingivalis* elicited a similar chemokine profile by the cells, suggested to be attributed to the effect of gingipains or LPS [11]. *Porphyromonas gingivalis* has also been shown to induce the expression of CCL2 in endothelial cells [18] and CCL2 and CCL5 in bone-marrow derived cells [19]. Evidence from clinical biopsies demonstrates that CCL2 and CCL5 gene expressions are up-regulated in aggressive and chronic periodontitis [20]. Therefore, the progressive induction of chemokines by *P. gingivalis* could facilitate the recruitment of inflammatory cells on site, and corroborate the establishment of chronic inflammation.

Among the interleukin (IL) family of cytokines, the pro-inflammatory IL-1α and IL-6 were increased at 24 h by 6.5-fold and 23.2-fold, respectively. However, the expression of IL-15, a cytokine that regulates T-cell and natural killer cell activation and proliferation, was down-regulated by 2.3-fold at this time-point. In support of these findings, *P. gingivalis* has been previously shown to stimulate of IL-6 mRNA [8], [21] and protein expression [22]. Earlier work has also confirmed the induction of IL-1α production in human monocytes in response to *P. gingivalis*, albeit to a weaker extent that other putative periodontal pathogens [23]. Both IL-1α and IL-6 are considered as key cytokines in the stimulation of mechanisms of bone resorption [24].

Further data on the regulation of cytokines and mediators of bone resorption is presented. Macrophage-colony stimulating factor (M-CSF) 1 is a cytokine required for survival, proliferation and differentiation of hematopoeitic cells into osteoclasts. M-CSF 1 expression was increased by 2.7-fold in response *P. gingivalis*, after 6 h. Additionally, RANKL expression, the key stimulator of osteoclast differentiation, was up-regulated at 24 h by 1.9 fold (Table 2, Cytokines), whereas the expression of OPG, the natural inhibitor of RANKL, and subsequently of bone resorption, was down-regulated by 4.0-fold as early as 6 h (Table 2, Receptor genes). These changes in RANKL and OPG gene expression are well in line with earlier results in this experimental system, which also demonstrated concomitant changes on the protein level [9], [10]. *P. gingivalis* has also been shown to up-regulate RANKL and down-regulate OPG gene expression in periodontal ligament cells and gingival fibroblasts [25]. Activated B-cells and T- cells can also express RANKL [26] and *P. gingivalis* stimulates further its expression in T-cells [27]. Prostaglandin (PG)E_2_ is a major inflammatory mediator of bone resorption, which can also induce the expression of RANKL expression [9], [10], [27]. It is well established that PGE_2_ is involved in the pathogenesis of periodontitis [28] and the stimulation of bone resorption [29]. Key enzymes involved in PGE_2_ synthesis are COX-2 and prostaglandin E synthase (PTGES). The gene expression of COX-2 was increased in response to *P. gingivalis* by 3.2-fold and 2.3-fold at 6 h and 24 h respectively, whilst PTGES expression was increased by 3.2-fold at 6 h. These findings are in line with earlier studies demonstrating the up-regulation of PGE_2_ in various cell types by *P. gingivalis* [8], [27], [30]. Collectively, this microarray data confirms that *P. gingivalis* enhances the gene expression of a number of inflammatory mediators, providing favourable conditions for the stimulation of osteoclastogenesis and bone resorption.

Haptoglobin (Hp), a protein that binds haemoglobin in plasma, is associated with acute phase response. It prevents heme-mediated oxidative damage to internal organs upon haemolysis during pathologic conditions, such as bacterial infections [31]. In the present experimental system, *P. gingivalis* caused up-regulation of Hp expression by 2.6-fold and 7.5-fold, at 6 h and 24 h, respectively. The expression of hemopexin (Hpx), a protein that binds heme, was also up-regulated by 7-fold at 24 h. Systemic Hp levels are shown to be higher in individuals with periodontitis, compared to periodontally healthy ones [32]. Therefore, the up-regulation of Hp expression by *P. gingivalis* may theoretically contribute to the presence of systemic inflammatory response. Another important consideration is that *P. gingivalis* utilizes hemin to receive iron for its growth, a process inhibited by host iron-sequestering (binding) proteins, including Hp and Hpx, which form complexes with haemoglobin and heme, respectively, within blood plasma, thereby denying iron to bacteria. As *P. gingivalis* can grow in the presence of these complexes, it has been suggested that it may possess a mechanism through which it is able to utilize iron from them [33]. In fact, the capacity of *P. gingivalis* Lys-specific gingipain to degrade Hp and Hpx with subsequent release of hemin from these complexes could constitute a mechanism that facilitates its growth [34]. Therefore, the up-regulation of Hp and Hpx by *P. gingivalis* may enhance binding of iron from hemin, which is required within its nutrient milieu.

Interestingly, within this category of inflammation-related genes were also two among the most regulated ones, namely lipocalin 2 (LCN2) and serum amyloid A3 (SAA3). LCN2 expression exhibited a dramatic increase in response to *P. gingivalis*, which was 33.6-fold and 354.8-fold at 6 h and 24 h, respectively. SAA3 expression was also strongly up-regulated in response to *P. gingivalis* by 144.8-fold and 143.9-fold at 6 h and 24 h, respectively (Table 2, Inflammation genes). LCN genes encode for small glycoproteins, which arise as part of the acute phase response, with involvement in the innate immune response to bacterial infection. LCN2 exerts antimicrobial effects by sequestering bacterial siderophores and inhibiting the growth of iron-dependent bacteria [35]. Although, *P. gingivalis* does not use sidephores to sequester iron [36], but rather haemophores [37], [38] the dramatic up-regulation of LCN2 expression may still constitute an iron-sequesteing based antimicrobial response, aiming to limit its growth. The SAA family of proteins are also involved in the acute phase response, consisting of four isoforms. SAA3 is an active isoform in mice [39], with 70% amino-acid sequence homology and overlapping function to human SAA1 [40]. The strong up-regulation of SAA3 transcription in response to *P. gingivalis* in the present experimental system is in line with earlier work on murine ST2 stromal cells challenged with another *P. gingivalis* strain, demonstrating an 11.5-fold after 6 h of challenge [11]. Sustained production of SAA proteins can lead to amyloidosis, through fibril deposition, and consequently to chronic inflammatory conditions [41]. A link may exist between amyloidosis and periodontal disease, where one condition may predispose to, or aggravate, the other [42], [43]. Interestingly, periodontitis patients exhibit higher SAA blood serum levels than healthy individuals [44]. Therefore, SAA protein levels could serve as a potential biomarker for periodontal disease progression, or could constitute a potential molecular link between periodontal diseases and systemic inflammatory conditions. In this line, the involvement of SAA3 requires further clinical investigation.

### Immune System

*Porphyromonas gingivalis* also stimulated the expression of a number of immune related genes by the cells (Table 2, Immune related genes). Cathelicidin antimicrobial peptide (CAMP) expression, a protein that exhibits antibacterial activity, was up-regulated by 9.4-fold at 24 h. CAMP has a role in the endosomal degradation of the bacterial cell membrane during phagocytosis, and can also bind to LPS, thus inhibiting its biological activity [45]. Mesenchymal stromal cells can also secrete CAMP in response to Gram-negative bacteria, which can in turn inhibit their growth [46]. Moreover, CAMP can inhibit host pro-inflammatory responses elicited by *P. gingivalis*, or its LPS and fimbriae. Although the present data on CAMP could indicate a potential antimicrobial response of *P. gingivalis*, it has also been shown that this species is resistant to the anti-microbial actions of CAMP [47], [48].

Genes encoding for complement proteins C1s, C3, C4B and complement factor B (CFB) were also up-regulated at 24 h. It is well known that *P. gingivalis* is also able to degrade complement factors [49], [50]. In particular, its gingipains can either degrade or, at low concentrations, activate complement factors [51], and Arg-X and Lys-X gingipain mutants have increased susceptibility to complement killing, compared to wild-type *P. gingivalis* [52]. The induction of the expression of complement factors by *P. gingivalis* suggests that cells are mounting an immune response aimed at clearing bacterial infection. Nevertheless, there is recent evidence that *P. gingivalis* manipulates innate immunity by controlling the cross-talk between complement factors and the host [53]–[57] Therefore, the induction of complement-associated genes observed in this experimental model may well be an attempt of *P. gingivalis* to confuse the innate immune host responses.

Other strongly up-regulated genes linked with innate immunity were Pentraxin 3 (PTX3), Spondin 2 (SPON2) and Ubiquitin D (UBD). A notable up-regulation was observed in the case of PTX3, which was 11.9-fold and 32.0-fold, at 6 h and 24 h, respectively. PTX3 protein is induced in response to stimulation with LPS or inflammatory cytokines, and is involved in a variety of innate immunity and inflammatory processes [58]. With regards to periodontitis, PTX3 levels are associated with the severity of the disease [59], and diseased sites foster higher PTX3 levels compared to healthy ones [60]. In line with the present observations, *P. gingivalis* LPS and fimbriae have been shown to induce PTX3 expression in macrophages [61].

### Growth Factors

The transcription of some growth factors was also affected by *P. gingivalis* challenge. In particular, a down-regulation was evident in the expressions of bone morphogenic protein (BMP)4 and transforming growth factor β-3 (TGFβ3). BMP4 is a key gene in the process of stimulating osteoblastic differentiation [62], and this was down-regulated by 2.2-fold at 6 h. TGFβ3 has crucial roles in osteogenic and chondrogenic differentiation by bone marrow stromal cells and its expression was decreased by 2.1-fold and 2.5-fold, at 6 h and 24 h, respectively, in the present experimental system. Collectively, these effects of *P. gingivalis* suggest a potential inhibition of the osteogenic and chondrogenic differentiation capacities of the studied cells.

On the contrary, the gene expressions of vascular endothelial growth factor (VEGF) and hepatocyte growth factor (HGF) were up-regulated after 24 h of challenge with *P. gingivalis* by 2.8-fold, and 2.3-fold, respectively (Table 2, Growth factors genes). VEGF is a potent inducer of angiogenesis and increased vascular permeability. Increased vascularisation, swelling and oedema are characteristic of periodontal inflammation. To this extent, several studies implicate VEGF in the progression of periodontitis, since increased VEGF levels were observed in diseased gingival tissue [63] or GCF from diseased sites [64], compared to healthy ones. In line with the present findings, it has been shown that *P. gingivalis* vesicles and outer membrane proteins increased VEGF expression in human gingival fibroblasts [65]. Collectively, these findings suggest a mechanism by which *P. gingivalis* can promote angiogenesis and vascular permeability during the progress of periodontitis. Moreover, the increased vascularisation can subsequently lead to increased hemin concentrations, which are much requited for the growth of *P. gingivalis*.

### Proteases

The regulation of genes encoding for some proteases and other functionally similar proteins was also affected. Two A Disintegrin And Metalloproteinases (ADAM) proteases were differentially expressed. ADAM domain 12 (ADAM12) was down-regulated at 6 h by 2.4- fold, whereas ADAM with thrombospondin (ADAMTS7) was up-regulated at 24 h by 2.1-fold (Table 2, Protease and related genes). Another member of the ADAM family, ADAM17, was previously shown to be up-regulated in T-cells, in response to *P. gingivalis* [66]. The expression levels of cysteine and serine proteases were also affected. Cysteine protease cathepsin L (CTSL) was up-regulated at 6 h whilst elastase 1 (ELA1), a serine protease, was down-regulated at both 6 h and 24 h. By degrading extracellular matrix components, CTSL may be involved in the metabolic turnover of bone [67]. Elastases are capable of cleaving collagen and elastin [68], with substantial evidence to support a role a neutrophil elastase ELA2 in the pathogenesis of periodontitis [69]–[71] The down-regulation of ELA1 in the present experimental system, may suggest a previously uncharacterised role in the pathogenesis of periodontal disease.

Interestingly, the expression of secretory leukocyte peptidase inhibitor (SLPI), a serine proteinase inhibitor of elastase and capthepsin G [72], was increased by 4.9-fold and 19.5-fold at 6 h and 24 h, respectively (Table 2, Protease and related genes). SLPI levels from periodontitis patients were found to be lower than healthy individuals in GCF [73] or gingival tissue [74], compared to healthy individuals. A decrease in SLPI GCF levels is associated with high *P. gingivalis* levels in dental plaque [73], but this could be attributed to the inhibition of the protective effect of SLPI by the Arg-X gingipain [75]. The serine protease inhibitor (SERPIN) gene family, comprises of several serine and selected cysteine proteinase inhibitors that inhibit serine proteases and some cysteine proteases, such as elastase and cathepsin L [76]. Several SERPIN family genes were up-regulated in response to *P. gingivalis* challenge, in the present experimental system. Serpin b6 and Serpin b9 expressions were increased at 24 h, whereas Serpin 3f and Serpin 3 g expressions were increased at both 6 h and 24 h (Table 2, Proteases and related genes). The up-regulation of these Serpins could be a defence mechanism against *P. gingivalis*, in order to inhibit the activity of cysteine proteinases (gingipains).

Matrix metalloproteinases (MMPs) are zinc-dependent endopeptidases that are normally involved in tissue and extracellular matrix remodelling, and their over-expression leads to degradation of collagen and fibronectin, contributing to periodontal tissue and bone destruction [28]. In response to *P. gingivalis* challenge, the expression of several MMPs was up-regulated. MMP17 was up-regulated at 6 h, whereas MMP12, MMP3 and MMP9 were up-regulated at 24 h. In particular, MMP3 was increased by a marked 35.8-fold at 24 h, whereas MMP13 was up-regulated at both 6 h and 24 h, by 9.3-fold and 8.4 fold, respectively. These findings may not be surprising, as several studies demonstrated that *P. gingivalis* are capable of simulating the production of MMPs in a variety of cell types, including periodontal ligament, human dental pulp, and gingival fibroblast cells [77]–[80]. Tissue inhibitor of metalloproteinase 1 (TIMP1), an MMP inhibitor, was also up-regulated at 6 h by 2.1-fold. A large body of evidence demonstrates elevated levels of MMPs in periodontal disease compared to health [81]–[85]. Furthermore, there a positive correlation is revealed clinically between the levels of *P. gingivalis* detected in active sites and MMP13 levels [86]. The expression of TIMP1, an MMP inhibitor, was also up-regulated by *P. gingivalis* in the present experimental system. To this extent, *P. gingivalis* is able to degrade TIMP1, with gingipains suggested as the responsible virulence factor for this effect [80], [87].

### Receptors

The transcription of a number of receptors was increased in response to *P. gingivalis* challenge (Table 2, Receptor genes). Cluster of differentiation 14 (CD14), a major receptor required for the activation of toll-like receptor complexes in response to LPS, was up-regulated by 2.1-fold at 24 h, whereas Toll like receptor (TLR)-2 was up-regulated by 3.7-fold and 12.6-fold, at 6 h and 24 h, respectively. A number of genes associated with the TLR-2 receptor complex, including CD14, LBP and TLR-2, were uniformly up-regulated by *P. gingivalis* in the present experimental system. Conversely, the transcription of MD-2, the co-receptor of TLR-4 was down-regulated in the current work. The expression of two signaling molecules involved in downstream TLR signaling was also affected. IRAK3, a negative regulator of TLR signaling, and MYD88, a critical signaling component of TLR signaling, were also increased. *P. gingivalis* fimbriae or its LPS are recognized by either TLR-2 [88]–[90] TLR-4 [91], [92] in various cell types. In agreement with the present findings, earlier work has shown that *P. gingivalis* induces TLR-2 expression [93], [94]. Interestingly, cytokine inflammatory response and alveolar bone loss was considerably reduced in TLR-2 deficient mice, after infection with *P. gingivalis* [95]. Taken together, TLR-2 is a key signaling receptor in the recognition of *P. gingivalis* by host cells, and through its down-stream pathway it may contribute to the overall inflammatory response.

### Signal Transduction and Transcription Factor Genes

Over the course of *P. gingivalis* challenge, the expression of several genes involved in signal transduction was increased. As bone marrow stromal cells can differentiate into a number of cell types, it is not surprising that a broad array of transcription factors have been affected. A total of 25 such genes were regulated in the cells in response to *P. gingivalis* challenge (Table 2, Signal transduction genes/Transcription factor and inhibitor genes). The following presentation and discussion is focused on a few such genes with relevance to the pathogenesis of periodontal disease.

Janus kinase 2 (JAK2), a protein tyrosine kinase, was up-regulated at 24 h by 6.9-fold. Moreover, the associated signal transducer and activator of transcription 3 (STAT3) transcription factor, as well as the suppressor of cytokine signalling 3 (SOCS3), a negative regulator of cytokines that signal through the JAK/STAT pathways, were also up-regulated. The JAK/STAT pathway is activated when cytokines or growth factors bind to JAK-associated receptors, which become trans-phosphorylated [96], in turn allowing STAT transcription factors to be phosphorylated and initiate gene transcription [97]. Live *P. gingivalis* has been shown to activate STAT3 and JAK1 in gingival epithelial cells [98], and to induce STAT1, STAT2 and SOCS1 gene expression in stromal cells [11]. *P. gingivalis* FimA up-regulated, whereas LPS down-regulated, STAT3 expression in macrophages [99]. Interestingly, STAT3 regulates RANKL expression in osteoblasts, with implications in the initiation of mechanisms of bone resorption [100]–[102].

CCAAT/enhancer-binding protein beta (C/EBPβ), a transcription factor involved in osteoblast differentiation *in vivo* [103] and the induction of RANKL gene expression in osteoblast-like cells [104], was also up-regulated in response to *P. gingivalis*. Thus, C/EBPβ up-regulation may contribute to the induction of RANKL by *P. gingivalis*, hinting for pathways additional to the recently identified p38 MAPK [10].

The expression of hairy-related transcription factor 1 (HEY1), which has a negative effect on osteoblastic differentiation [105], was increased after 6 h of challenge with *P. gingivalis*. As it has recently been shown that HEY1 is involved in *P. gingivalis* LPS-mediated inhibition of osteoblastic differentiation [106], the present findings suggest that *P. gingivalis* may impair osteoblast differentiation by bone marrow stromal cells, mediated by HEY1.

Components of the NF-κB complex, NF-κB 1, NF-κB 2, v-rel reticuloendotheliosis viral oncogene homolog (REL) A and REL-B were all up-regulated at 24 h (Table 2, Transcription factor genes). The NF-kB is a key pathway in the activation of genes involved in immunity and inflammation, and its deregulation can lead to pathological conditions such as cancer and chronic inflammatory disorders [107]. Indeed, NF-κB gingival tissue expression is higher in chronic periodontitis, compared to health [108]. Pro-inflammatory cytokines, such as IL-1β, TNF-α, as well as bacterial LPS are common inducers of NF-kB [109]. NF-kB complexes are normally inactive and stabilized in the cytoplasm through the association of bound IkB proteins. Phosphorylation of these proteins by IkB kinases lead to their dissociation from the NF-KB complex, whereby the latter are then free to translocate to the nucleus, initiating gene transcription [107]. Interestingly, the expression of a number of NF-κB inhibitors (BCL3, NF- κBIA, NF-κBIE, NF-κBIZ, TNFAIP3 and TNIP1) as well as kinases responsible for their phosphorylation (IκBKE and IκBKG) was also up-regulated. NF-κBIZ in particular demonstrated a marked 11.2-fold and 9.6-fold up-regulation, at 6 h and 24 h, respectively. The present findings are in agreement with previous studies demonstrating a central role of NF-κB in the pro-inflammatory responses to *P. gingivalis* in various cell types including fibroblasts, stromal cells, gingival epithelial cells, osteoblasts, cementoblasts and endothelial cells [11], [90], [93], [110]–[112].

Components of the Activator Protein (AP)-1 transcription factor complex jun B proto-oncogene (JUNB) and FBJ murine osteosarcoma viral oncogene homolog (c-Fos), were also increased at 6 h and 24 h. These findings are in agreement with earlier work demonstrating that *P. gingivalis* is able to activate the AP-1 transcription factor in osteoblasts, HUVEC’s and oral epidermoid cells [8], [110], [113].

Taken together, the present findings using microarray technology support the notion that *P. gingivalis* can activate multiple intracellular signalling pathways and transcription factors in a complex manner.

### Validation of Selected Microarray Data by qPCR

To interpret the results obtained from the microarray dataset, it was important to validate representative items of the data, using other mRNA detection assays, in this case qPCR. Therefore RANKL, OPG and COX-2 gene expressions were assayed, as these genes were previously shown to be regulated in the present experimental system [9], [10]. A comparable trend of expression between the gene microarray and the qPCR results was indeed confirmed (Table 3). To further validate the data obtained by microarray analysis, the expression of acute phase inflammation-associated genes LCN2 and SAA3 was also investigated by qPCR, as these genes displayed the greatest differential regulation in the microarray dataset. As indicated (Table 3) the trend of LCN2 and SAA3 up-regulation at 6 h and 24 h was also confirmed by qPCR, although the magnitude of was markedly higher by qPCR. This could be attributed to that the dynamic range at which mRNA expression can be measured is relatively limited by the microarray technology, whereas qPCR assays are particularly sensitive in this respect [114]. As the –fold changes in SAA3 expression were among the highest measured, and these were similar among the two time-points, a plausible explanation for the quantitative discrepancy between the two assays is that the microarray analysis measurements may have been saturated at this high detection range.

**Table 3:** Validation of microarray data by qPCR analysis.

Fold changes in expression of selected genes by microarray					
	COX-2	LCN2	OPG	RANKL	SAA3
6 h	3.2***	33.6***	−4.0***		144.8***
24 h	2.3***	354.8***		1.9***	143.9***
**Fold changes in expression of selected genes by qPCR**					
	**COX-2**	**LCN2**	**OPG**	**RANKL**	**SAA3**
6 h	10.1**	352.2***	−4.0*	5.4**	4817.0***
24 h	5.7***	4097.0***	−1.2	15.3*	95296.4***

Five of regulated genes were representatively selected for validation of their expression levels by qPCR, in comparison to the microarray. These were namely COX-2, LCN2, OPG, RANKL and SAA3. The values represent fold-changes of gene expression levels in response to *P. gingivalis*, in relation to control, by microarray analysis (A), or by qPCR analysis (B). The minus values (also in bold letters) indicate down-regulation. When a fold-change value is missing in one of the two time-points, the regulation of gene expression at that time-point was less that 2-fold. *p<0.05,

** *p*<0.01,

*** *p*<0.001.

### Conclusions

This *in vitro* study employed gene microarray technology to investigate in a temporal manner the global transcriptional events in bone marrow stromal cells, in response to the major periodontal pathogen *Porphyromonas gingivalis*. These cells have osteogenic potential and, once exiting the bone marrow, they can differentiate into bone forming osteoblasts, which are crucial for the maintenance of homeostasis in the periodontal tissues. Thus, by interfering with the transcriptional regulation of these cells, *P. gingivalis* may affect the homeostatic capacity of the tissues. The findings demonstrate time-dependent responses of the cells to *P. gingivalis* challenge. The early (6 h) responses are characterised by enhanced expression of genes associated with inhibition of cell cycle, induction of apoptosis and loss of structural integrity, which can be collectively perceived as a hindrance of the basic cell functions. The later (24 h) responses are characterised by induction of cytokines, chemokines, mediators of connective tissue and bone destruction, and suppression of regulators of osteogenic differentiation. All these effects denote loss of the anabolic capacity by the cells, switching into a catabolic phenotype that favours inflammatory tissue destruction. However, antimicrobial protein expression is also enhanced, potentially as a host protective mechanism to tackle the bacterial challenge. Moreover, the expression of genes associated with vascularisation and heme-binging capacity is also increased. This effect, apart from being a further inflammatory trait, could be induced by *P. gingivalis* as a tactique to gain access to much needed nutrients. In conclusion, *P. gingivalis* induces a diverse transcriptional profile response in bone marrow stromal cells, with evident implications in the deregulation of tissue homeostasis and the pathogenesis of periodontal disease.

## Supporting Information

Table S1 List of genes regulated after 6 h in response to *Porphyromonas gingivalis.*
(DOC)

Table S2 List of genes regulated after 24 h in response to *Porphyromonas gingivalis.*
(DOC)

## References

[1] Yang H-W, Huang Y-F, Chou M-Y (2004) Occurrence of Porphyromonas gingivalis and Tannerella forsythensis in Periodontally Diseased and Healthy Subjects. Journal of Periodontology 75: 1077–1083. 15455734 10.1902/jop.2004.75.8.1077

[2] Van Winkelhoff AJ, Loos BG, Van Der Reijden WA, Van Der Velden U (2002) Porphyromonas gingivalis, Bacteroides forsythus and other putative periodontal pathogens in subjects with and without periodontal destruction. Journal of Clinical Periodontology 29: 1023–1028. 12472995 10.1034/j.1600-051x.2002.291107.x

[3] Hajishengallis G (2009) Porphyromonas gingivalis-host interactions: open war or intelligent guerilla tactics? Microbes and Infection 11: 637–645. 19348960 10.1016/j.micinf.2009.03.009 PMC2704251

[4] Bostanci N, Belibasakis GN (2012) Porphyromonas gingivalis: an invasive and evasive opportunistic oral pathogen. FEMS Microbiology Letters 333: 1–9. 22530835 10.1111/j.1574-6968.2012.02579.x

[5] Hajishengallis G, Krauss JL, Liang S, McIntosh ML, Lambris JD (2012) Advances in Experimental Medicine and Biology, 1. Current Topics in Innate Immunity II 946: 69–85. 10.1007/978-1-4614-0106-3_5 PMC3214273 21948363

[6] Lamont RJ, Jenkinson HF (1998) Life Below the Gum Line: Pathogenic Mechanisms of Porphyromonas gingivalis. Microbiol Mol Biol Rev 62: 1244–1263. 9841671 10.1128/mmbr.62.4.1244-1263.1998 PMC98945

[7] Thies RS, Bauduy M, Ashton BA, Kurtzberg L, Wozney JM, et al.. (1992) Recombinant human bone morphogenetic protein-2 induces osteoblastic differentiation in W-20–17 stromal cells. 1318–1324. 10.1210/endo.130.3.1311236 1311236

[8] Okahashi N, Inaba H, Nakagawa I, Yamamura T, Kuboniwa M, et al (2004) Porphyromonas gingivalis Induces Receptor Activator of NF-{kappa}B Ligand Expression in Osteoblasts through the Activator Protein 1 Pathway. Infect Immun 72: 1706–1714. 14977979 10.1128/IAI.72.3.1706-1714.2004 PMC356028

[9] Reddi D, Bostanci N, Hashim A, Aduse-Opoku J, Curtis MA, et al (2008) Porphyromonas gingivalis regulates the RANKL-OPG system in bone marrow stromal cells. Microbes and Infection 10: 1459–1468. 18789397 10.1016/j.micinf.2008.08.007

[10] Reddi D, Brown SJ, Belibasakis GN (2011) Porphyromonas gingivalis induces RANKL in bone marrow stromal cells: Involvement of the p38 MAPK. Microbial Pathogenesis 51: 415–420. 21939752 10.1016/j.micpath.2011.09.001

[11] Ohno T, Okahashi N, Kawai S, Kato T, Inaba H, et al (2006) Proinflammatory gene expression in mouse ST2 cell line in response to infection by Porphyromonas gingivalis. Microbes and Infection 8: 1025–1034. 16476562 10.1016/j.micinf.2005.10.021

[12] Ohno T, Okahashi N, Morisaki I, Amano A (2008) Signaling pathways in osteoblast proinflammatory responses to infection by Porphyromonas gingivalis. Oral Microbiology and Immunology 23: 96–104. 18279176 10.1111/j.1399-302X.2007.00393.x

[13] Imatani T, Kato T, Okuda K, Yamashita Y (2004) Histatin 5 inhibits apoptosis in human gingival fibroblasts induced by porphyromonas gingivalis cell-surface polysaccharide. European Journal Of Medical Research 9: 528–532. 15649864

[14] Roth GA, Ankersmit HJ, Brown VB, Papapanou PN, Schmidt AM, et al (2007) Porphyromonas gingivalis infection and cell death in human aortic endothelial cells. FEMS Microbiology Letters 272: 106–113. 17459112 10.1111/j.1574-6968.2007.00736.x

[15] Stathopoulou P, Galicia J, Benakanakere M, Garcia C, Potempa J, et al (2009) Porphyromonas gingivalis induce apoptosis in human gingival epithelial cells through a gingipain-dependent mechanism. BMC Microbiology 9: 107. 19473524 10.1186/1471-2180-9-107 PMC2692854

[16] Kato T, Tsuda T, Inaba H, Kawai S, Okahashi N, et al (2008) Porphyromonas gingivalis gingipains cause G1 arrest in osteoblastic/stromal cells. Oral Microbiology and Immunology 23: 158–164. 18279184 10.1111/j.1399-302X.2007.00405.x

[17] Inaba H, Kuboniwa M, Bainbridge B, Yilmaz Ö, Katz J, et al (2009) Porphyromonas gingivalis invades human trophoblasts and inhibits proliferation by inducing G1 arrest and apoptosis. Cellular Microbiology 11: 1517–1532. 19523155 10.1111/j.1462-5822.2009.01344.x PMC2766574

[18] Kang I-C, Kuramitsu HK (2002) Induction of monocyte chemoattractant protein-1 by Porphyromonas gingivalis in human endothelial cells. FEMS Immunology & Medical Microbiology 34: 311–317. 12443832 10.1111/j.1574-695X.2002.tb00639.x

[19] Jiang Y, Graves DT (1999) Periodontal Pathogens Stimulate CC-Chemokine Production by Mononuclear and Bone-Derived Cells. Journal of Periodontology 70: 1472–1478. 10632523 10.1902/jop.1999.70.12.1472

[20] Garlet GP, Martins W, Ferreira BR, Milanezi CM, Silva JS (2003) Patterns of chemokines and chemokine receptors expression in different forms of human periodontal disease. Journal of Periodontal Research 38: 210–217. 12608917 10.1034/j.1600-0765.2003.02012.x

[21] Zhou Q, Desta T, Fenton M, Graves DT, Amar S (2005) Cytokine Profiling of Macrophages Exposed to Porphyromonas gingivalis, Its Lipopolysaccharide, or Its FimA Protein. Infection and Immunity 73: 935–943. 15664935 10.1128/IAI.73.2.935-943.2005 PMC547047

[22] Laflamme C, Rouabhia M, Le X (2009) Porphyromonas gingivalis decreases osteoblast proliferation through IL-6-RANKL/OPG and MMP-9/TIMPs pathways. 141–149 p. 10.4103/0970-9290.52884 19553712

[23] Bostanci N, Allaker R, Johansson U, Rangarajan M, Curtis MA, et al (2007) Interleukin-1alpha; stimulation in monocytes by periodontal bacteria: antagonistic effects of Porphyromonas gingivalis. Oral Microbiology and Immunology 22: 52–60. 17241171 10.1111/j.1399-302X.2007.00322.x

[24] Liu Y-CG, Lerner UH, Teng Y-TA (2010) Cytokine responses against periodontal infection: protective and destructive roles. Periodontology 2000 52: 163–206. 20017801 10.1111/j.1600-0757.2009.00321.x

[25] Belibasakis GN, Bostanci N, Hashim A, Johansson A, Aduse-Opoku J, et al (2007) Regulation of RANKL and OPG gene expression in human gingival fibroblasts and periodontal ligament cells by Porphyromonas gingivalis: A putative role of the Arg-gingipains. Microbial Pathogenesis 43: 46–53. 17448630 10.1016/j.micpath.2007.03.001

[26] Kawai T, Matsuyama T, Hosokawa Y, Makihira S, Seki M, et al (2006) B and T Lymphocytes Are the Primary Sources of RANKL in the Bone Resorptive Lesion of Periodontal Disease. The American Journal of Pathology 169: 987–998. 16936272 10.2353/ajpath.2006.060180 PMC1698808

[27] Belibasakis GN, Reddi D, Bostanci N (2011) Porphyromonas gingivalis Induces RANKL in T-cells. Inflammation 34: 133–138. 20446027 10.1007/s10753-010-9216-1

[28] Offenbacher S (1996) Periodontal diseases: pathogenesis. Annals of Periodontology 1: 821–878. 9118282 10.1902/annals.1996.1.1.821

[29] Noguchi K, Ishikawa I (2007) The roles of cyclooxygenase-2 and prostaglandin E2 in periodontal disease. Periodontology 2000 43: 85–101. 17214837 10.1111/j.1600-0757.2006.00170.x

[30] Choi B-K, Moon S-Y, Cha J-H, Kim K-W, Yoo Y-J (2005) Prostaglandin E2 Is a Main Mediator in Receptor Activator of Nuclear Factor-kappaB Ligand-Dependent Osteoclastogenesis Induced by Porphyromonas gingivalis, Treponema denticola, and Treponema socranskii. Journal of Periodontology 76: 813–820. 15898943 10.1902/jop.2005.76.5.813

[31] Tolosano E, Fagoonee S, Hirsch E, Berger FG, Baumann H, et al (2002) Enhanced splenomegaly and severe liver inflammation in haptoglobin/hemopexin double-null mice after acute hemolysis. Blood 100: 4201–4208. 12393471 10.1182/blood-2002-04-1270

[32] Ebersole JL, Machen RL, Steffen MJ, DE W (1997) Systemic acute-phase reactants, C-reactive protein and haptoglobin, in adult periodontitis. Clinical and experimental immunology 107: 347–352. 9030874 10.1111/j.1365-2249.1997.270-ce1162.x PMC1904587

[33] Bramanti TE, Holt SC (1991) oles of porphyrins and host iron transport proteins in regulation of growth of Porphyromonas gingivalis W50. Journal of Bacteriology 173: 7330–7339. 1657888 10.1128/jb.173.22.7330-7339.1991 PMC209241

[34] Sroka A, Sztukowska M, Potempa J, Travis J, Genco CA (2001) Degradation of Host Heme Proteins by Lysine- and Arginine-Specific Cysteine Proteinases (Gingipains) ofPorphyromonas gingivalis. Journal of Bacteriology 183: 5609–5616. 11544223 10.1128/JB.183.19.5609-5616.2001 PMC95452

[35] Flo TH, Smith KD, Sato S, Rodriguez DJ, Holmes MA, et al (2004) Lipocalin 2 mediates an innate immune response to bacterial infection by sequestrating iron. Nature 432: 917–921. 15531878 10.1038/nature03104

[36] Olczak T, Simpson W, Liu X, CA G (2005) Iron and heme utilization in Porphyromonas gingivalis. FEMS Microbiol Rev 29: 119–144. 15652979 10.1016/j.femsre.2004.09.001

[37] Wojaczyński J, Wójtowicz H, Bielecki M, Olczak M, Smalley JW, et al (2011) Iron(III) mesoporphyrin IX and iron(III) deuteroporphyrin IX bind to the Porphyromonas gingivalis HmuY hemophore. Biochemical and Biophysical Research Communications 411: 299–304. 21740890 10.1016/j.bbrc.2011.06.129

[38] Gao J-L, Nguyen K-A, Hunter N (2010) Characterization of a Hemophore-like Protein from Porphyromonas gingivalis. Journal of Biological Chemistry 285: 40028–40038. 20940309 10.1074/jbc.M110.163535 PMC3000985

[39] Kluve-Beckerman B, Drumm ML, MD B (1991) Nonexpression of the human serum amyloid A three (SAA3) gene. DNA and Cell Biology 10: 651–665. 1755958 10.1089/dna.1991.10.651

[40] Reigstad CS, Bäckhed FF (2010) Microbial regulation of SAA3 expression in mouse colon and adipose tissue. Gut Microbes 1: 55–57. 21327118 10.4161/gmic.1.1.10514 PMC3035134

[41] Lachmann HJ, Goodman HJ, Gilbertson JA, Gallimore JR, Sabin CA, et al (2007) Natural history and outcome in systemic AA amyloidosis. The New England journal of medicine 356: 2361–2371. 17554117 10.1056/NEJMoa070265

[42] Khoury S, Dusek JJ, Anderson GB, Vigneswaran N (2004) Systemic amyloidosis manifesting as localized, severe periodontitis. The Journal of the American Dental Association 135: 617–623. 15202754 10.14219/jada.archive.2004.0250

[43] Cengiz MI, Wang HL, Levent Y (2010) Oral involvement in a case of AA amyloidosis: a case report. Journal of medical case reports 4: 200. 20591157 10.1186/1752-1947-4-200 PMC2911467

[44] Glurich I, Grossi S, Albini B, Ho A, Shah R, et al (2002) Systemic Inflammation in Cardiovascular and Periodontal Disease: Comparative Study. Clinical and Diagnostic Laboratory Immunology 9: 425–432. 11874889 10.1128/CDLI.9.2.425-432.2002 PMC119918

[45] Bals R, Wilson JM (2003) Cathelicidins - a family of multifunctional antimicrobial peptides. Cellular and Molecular Life Sciences 60: 711–720. 12785718 10.1007/s00018-003-2186-9 PMC11138611

[46] Krasnodembskaya A, Song Y, Fang X, Gupta N, Serikov V, et al (2010) Antibacterial Effect of Human Mesenchymal Stem Cells Is Mediated in Part from Secretion of the Antimicrobial Peptide LL-37. STEM CELLS 28: 2229–2238. 20945332 10.1002/stem.544 PMC3293245

[47] Altman H, Steinberg D, Porat Y, Mor A, Fridman D, et al (2006) In vitro assessment of antimicrobial peptides as potential agents against several oral bacteria. Journal of Antimicrobial Chemotherapy 58: 198–201. 16687459 10.1093/jac/dkl181

[48] Bachrach G, Altman H, Kolenbrander PE, Chalmers NI, Gabai-Gutner M, et al (2008) Resistance of Porphyromonas gingivalis ATCC 33277 to Direct Killing by Antimicrobial Peptides Is Protease Independent. Antimicrobial Agents and Chemotherapy 52: 638–642. 18086848 10.1128/AAC.01271-07 PMC2224744

[49] Wingrove JA, DiScipio RG, Chen Z, Potempa J, Travis J, et al (1992) Activation of complement components C3 and C5 by a cysteine proteinase (gingipain-1) from Porphyromonas (Bacteroides) gingivalis. Journal of Biological Chemistry 267: 18902–18907. 1527018

[50] Potempa J, Banbula A, Travis J (2000) Role of bacterial proteinases in matrix destruction and modulation of host responses. Periodontology 2000 24: 153–192. 11276866 10.1034/j.1600-0757.2000.2240108.x

[51] Popadiak K, Potempa J, Riesbeck K, Blom AM (2007) Biphasic effect of gingipains from Porphyromonas gingivalis on the human complement system. The Journal of Immunology 178: 7242–7250. 17513773 10.4049/jimmunol.178.11.7242

[52] Grenier D, Roy S, Chandad F, Plamondon P, Yoshioka M, et al (2003) Effect of Inactivation of the Arg- and/or Lys-Gingipain Gene on Selected Virulence and Physiological Properties of Porphyromonas gingivalis. Infection and Immunity 71: 4742–4748. 12874356 10.1128/IAI.71.8.4742-4748.2003 PMC166032

[53] Liang S, Krauss JL, Domon H, McIntosh ML, Hosur KB, et al (2010) The C5a Receptor Impairs IL-12-Dependent Clearance of Porphyromonas gingivalis and Is Required for Induction of Periodontal Bone Loss. The Journal of Immunology 186: 869–877. 21149611 10.4049/jimmunol.1003252 PMC3075594

[54] Krauss JL, Potempa J, Lambris JD, Hajishengallis G (2010) Complementary Tolls in the periodontium: how periodontal bacteria modify complement and Toll-like receptor responses to prevail in the host. Periodontology 2000 52: 141–162. 20017800 10.1111/j.1600-0757.2009.00324.x PMC2796596

[55] Guo Y, Nguyen K-A, Potempa J (2010) Dichotomy of gingipains action as virulence factors: from cleaving substrates with the precision of a surgeon’s knife to a meat chopper-like brutal degradation of proteins. Periodontology 2000 54: 15–44. 20712631 10.1111/j.1600-0757.2010.00377.x PMC2924770

[56] Wang M, Krauss JL, Domon H, Hosur KB, Liang S, et al.. (2010) Microbial Hijacking of Complement-Toll-Like Receptor Crosstalk. Sci Signal 3: ra11-. 10.1126/scisignal.2000697 PMC2824906 20159852

[57] Hajishengallis G, Liang S, Payne MA, Hashim A, Jotwani R, et al (2011) Low-Abundance Biofilm Species Orchestrates Inflammatory Periodontal Disease through the Commensal Microbiota and Complement. Cell host & microbe 10: 497–506. 22036469 10.1016/j.chom.2011.10.006 PMC3221781

[58] Ortega-Hernandez O-D, Bassi N, Shoenfeld Y, Anaya J-M (2009) The Long Pentraxin 3 and Its Role in Autoimmunity. Seminars in Arthritis and Rheumatism 39: 38–54. 18614204 10.1016/j.semarthrit.2008.03.006

[59] Pradeep AR, Kathariya R, Raghavendra NM, Sharma A (2011) Levels of Pentraxin-3 in Gingival Crevicular Fluid and Plasma in Periodontal Health and Disease. Journal of Periodontology 82: 734–741. 21080790 10.1902/jop.2010.100526

[60] Fujita Y, Ito H, Sekino S, Numabe Y (2011) Correlations between pentraxin 3 or cytokine levels in gingival crevicular fluid and clinical parameters of chronic periodontitis. Odontology: 1–7. 10.1007/s10266-011-0042-1 21932007

[61] Zhou Q, Amar S (2007) Identification of Signaling Pathways in Macrophage Exposed to Porphyromonas gingivalis or to Its Purified Cell Wall Components. The Journal of Immunology 179: 7777–7790. 18025224 10.4049/jimmunol.179.11.7777

[62] Katagiri T, Takahashi N (2002) Regulatory mechanisms of osteoblast and osteoclast differentiation. Oral Diseases 8: 147–159. 12108759 10.1034/j.1601-0825.2002.01829.x

[63] Johnson RB, Serio FG, Dai X (1999) Vascular Endothelial Growth Factors and Progression of Periodontal Diseases. Journal of Periodontology 70: 848–852. 10476891 10.1902/jop.1999.70.8.848

[64] Prapulla DV, Sujatha PB, Pradeep AR (2007) Gingival Crevicular Fluid VEGF Levels in Periodontal Health and Disease. Journal of Periodontology 78: 1783–1787. 17760549 10.1902/jop.2007.070009

[65] Suthin K, Matsushita K, Machigashira M, Tatsuyama S, Imamura T, et al (2003) Enhanced expression of vascular endothelial growth factor by periodontal pathogens in gingival fibroblasts. Journal of Periodontal Research 38: 90–96. 12558942 10.1034/j.1600-0765.2003.01646.x

[66] Bostanci N, Reddi D, Rangarajan M, Curtis MA, Belibasakis GN (2009) Porphyromonas gingivalis stimulates TACE production by T cells. Oral Microbiology and Immunology 24: 146–151. 19239642 10.1111/j.1399-302X.2008.00488.x

[67] Leto G, Crescimanno M, Flandina C, Sepporta M, Tumminello F (2011) Cathepsin L in Normal and Pathological Bone Remodeling. Clinical Reviews in Bone and Mineral Metabolism 9: 107–121.

[68] Dickinson DP (2002) Cysteine Peptidases of Mammals: Their Biological Roles and Potential Effects in the Oral Cavity and Other Tissues in Health and Disease. Critical Reviews in Oral Biology & Medicine 13: 238–275. 12090464 10.1177/154411130201300304

[69] Palcanis KG, Lariava IK, Wells BR, Suggs KA, Landis JR, et al (1992) Elastase as an Indicator of Periodontal Disease Progression. Journal of Periodontology 63: 237–242. 1573538 10.1902/jop.1992.63.4.237

[70] Armitage GC, Jeffcoat MK, Chadwick DE, Taggart EJ, Numabe Y, et al (1994) Longitudinal Evaluation of Elastase as a Marker for the Progression of Periodontitis. Journal of Periodontology 65: 120–128. 8158508 10.1902/jop.1994.65.2.120

[71] Jin LJ, Söder P-Ö, Åsman B, Söder B, Purlene A, et al (1995) Variations in crevicular fluid elastase levels in periodontitis patients on long-term maintenance. European Journal of Oral Sciences 103: 84–89. 7767710 10.1111/j.1600-0722.1995.tb00121.x

[72] Stetler G, Brewer MT, Thompson RC (1986) Isolation and sequence of a human gene encoding a potent inhibitor of leukocyte proteases. Nucleic Acids Research 14: 7883–7896. 3640338 10.1093/nar/14.20.7883 PMC311822

[73] Laugisch O, Schacht M, Guentsch A, Kantyka T, Sroka A, et al (2011) Periodontal pathogens affect the level of protease inhibitors in gingival crevicular fluid. Molecular Oral Microbiology 27: 45–56. 22230465 10.1111/j.2041-1014.2011.00631.x PMC3257822

[74] Kretschmar S, Yin L, Roberts F, London R, Flemmig TT, et al (2011) Protease inhibitor levels in periodontal health and disease. Journal of Periodontal Research 47: 228–235. 22029638 10.1111/j.1600-0765.2011.01425.x PMC3270203

[75] Into T, Inomata M, Kanno Y, Matsuyama T, Machigashira M, et al (2006) Arginine-specific gingipains from Porphyromonas gingivalis deprive protective functions of secretory leucocyte protease inhibitor in periodontal tissue. Clinical & Experimental Immunology 145: 545–554. 16907925 10.1111/j.1365-2249.2006.03156.x PMC1809709

[76] Silverman GA, Bird PI, Carrell RW, Church FC, Coughlin PB, et al (2001) The Serpins Are an Expanding Superfamily of Structurally Similar but Functionally Diverse Proteins. Journal of Biological Chemistry 276: 33293–33296. 11435447 10.1074/jbc.R100016200

[77] Nakata K, Yamasaki M, Iwata T, Suzuki K, Nakane A, et al (2000) Anaerobic Bacterial Extracts Influence Production of Matrix Metalloproteinases and Their Inhibitors by Human Dental Pulp Cells. Journal of endodontics 26: 410–413. 11199767 10.1097/00004770-200007000-00008

[78] Chang Y-C, Lai C-C, Yang S-F, Chan Y, Hsieh Y-S (2002) Stimulation of Matrix Metalloproteinases by Black-Pigmented Bacteroides in Human Pulp and Periodontal Ligament Cell Cultures. Journal of endodontics 28: 90–93. 11833696 10.1097/00004770-200202000-00010

[79] Pattamapun K, Tiranathanagul S, Yongchaitrakul T, Kuwatanasuchat J, Pavasant P (2003) Activation of MMP-2 by Porphyromonas gingivalis in human periodontal ligament cells. Journal of Periodontal Research 38: 115–121. 12608904 10.1034/j.1600-0765.2003.01650.x

[80] Zhou J, Windsor LJ (2006) Porphyromonas gingivalis affects host collagen degradation by affecting expression, activation, and inhibition of matrix metalloproteinases. Journal of Periodontal Research 41: 47–54. 16409255 10.1111/j.1600-0765.2005.00835.x

[81] Hernandez M, Valenzuela MA, Lopez-Otin C, Alvarez Js, Lopez JM, et al (2006) Matrix Metalloproteinase-13 Is Highly Expressed in Destructive Periodontal Disease Activity. Journal of Periodontology 77: 1863–1870. 17076612 10.1902/jop.2006.050461

[82] Hernández M, Martínez B, Tejerina JM, Valenzuela MA, Gamonal J (2007) MMP-13 and TIMP-1 determinations in progressive chronic periodontitis. Journal of Clinical Periodontology 34: 729–735. 17716308 10.1111/j.1600-051X.2007.01107.x

[83] Rai B, Kharb S, Jain R, Anand SC (2008) Biomarkers of periodontitis in oral fluids. Journal of Oral Science 50: 53–56. 18403884 10.2334/josnusd.50.53

[84] Balwant R, Jasdeep K, Rajnish J, Anand SC (2010) Levels of gingival crevicular metalloproteinases-8 and -9 in periodontitis. The Saudi Dental Journal 22: 129–131. 23960488 10.1016/j.sdentj.2010.04.006 PMC3723102

[85] Oyarzún A, Arancibia R, Hidalgo R, Peñafiel C, Cáceres M, et al (2010) Involvement of MT1-MMP and TIMP-2 in human periodontal disease. Oral Diseases 16: 388–395. 20233321 10.1111/j.1601-0825.2009.01651.x

[86] Silva N, Dutzan N, Hernandez M, Dezerega A, Rivera O, et al (2008) Characterization of progressive periodontal lesions in chronic periodontitis patients: levels of chemokines, cytokines, matrix metalloproteinase-13, periodontal pathogens and inflammatory cells. Journal of Clinical Periodontology 35: 206–214. 18269660 10.1111/j.1600-051X.2007.01190.x

[87] Grenier D, Mayrand D (2001) Inactivation of tissue inhibitor of metalloproteinases-1 (TIMP-1) by Porphyromonas gingivalis. FEMS microbiology letters 203: 161–164. 11583842 10.1111/j.1574-6968.2001.tb10835.x

[88] Asai Y, Ohyama Y, Gen K, Ogawa T (2001) Bacterial Fimbriae and Their Peptides Activate Human Gingival Epithelial Cells through Toll-Like Receptor 2. Infection and Immunity 69: 7387–7395. 11705912 10.1128/IAI.69.12.7387-7395.2001 PMC98826

[89] Nociti FH Jr, Foster BL, Barros SP, Darveau RP, Somerman MJ (2004) Cementoblast Gene Expression is Regulated by Porphyromonas gingivalis Lipopolysaccharide Partially via Toll-like Receptor-4/MD-2. J Dent Res 83: 602–607. 15271967 10.1177/154405910408300804

[90] Nemoto E, Darveau RP, Foster BL, Nogueira-Filho GR, Somerman MJ (2006) Regulation of Cementoblast Function by P. gingivalis Lipopolysaccharide via. TLR2: 733–738. 10.1177/154405910608500809 PMC2237885 16861291

[91] Reife RA, Coats SR, Al-Qutub M, Dixon DM, Braham PA, et al (2006) Porphyromonas gingivalis lipopolysaccharide lipid A heterogeneity: differential activities of tetra- and penta-acylated lipid A structures on E-selectin expression and TLR4 recognition. Cellular Microbiology 8: 857–868. 16611234 10.1111/j.1462-5822.2005.00672.x

[92] Sawada N, Ogawa T, Asai Y, Makimura Y, Sugiyama A (2007) Toll-like receptor 4-dependent recognition of structurally different forms of chemically synthesized lipid As of Porphyromonas gingivalis. Clinical & Experimental Immunology 148: 529–536. 17335558 10.1111/j.1365-2249.2007.03346.x PMC1941937

[93] Brozovic S, Sahoo R, Barve S, Shiba H, Uriarte S, et al (2006) Porphyromonas gingivalis enhances FasL expression via up-regulation of NFkB-mediated gene transcription and induces apoptotic cell death in human gingival epithelial cells. Microbiology 152: 797–806. 16514159 10.1099/mic.0.28472-0

[94] Hajishengallis G, Tapping RI, Harokopakis E, Nishiyama S-i, Ratti P, et al (2006) Differential interactions of fimbriae and lipopolysaccharide from Porphyromonas gingivalis with the Toll-like receptor 2-centred pattern recognition apparatus. Cellular Microbiology 8: 1557–1570. 16984411 10.1111/j.1462-5822.2006.00730.x

[95] Burns E, Bachrach G, Shapira L, Nussbaum G (2006) Cutting Edge: TLR2 Is Required for the Innate Response to Porphyromonas gingivalis: Activation Leads to Bacterial Persistence and TLR2 Deficiency Attenuates Induced Alveolar Bone Resorption. The Journal of Immunology 177: 8296–8300. 17142724 10.4049/jimmunol.177.12.8296

[96] Rawlings JS, Rosler KM, Harrison DA (2004) The JAK/STAT signaling pathway. Journal of Cell Science 117: 1281–1283. 15020666 10.1242/jcs.00963

[97] O'Shea JJ, Gadina M, Schreiber RD (2002) Cytokine Signaling in 2002: New Surprises in the Jak/Stat Pathway. Cell 109: S121–S131. 11983158 10.1016/s0092-8674(02)00701-8

[98] Mao S, Park Y, Hasegawa Y, Tribble GD, James CE, et al (2007) Intrinsic apoptotic pathways of gingival epithelial cells modulated by Porphyromonas gingivalis. Cellular Microbiology 9: 1997–2007. 17419719 10.1111/j.1462-5822.2007.00931.x PMC2886729

[99] Zhou Q, Amar S (2006) Identification of Proteins Differentially Expressed in Human Monocytes Exposed to Porphyromonas gingivalis and Its Purified Components by High-Throughput Immunoblotting. Infection and Immunity 74: 1204–1214. 16428770 10.1128/IAI.74.2.1204-1214.2006 PMC1360359

[100] O’Brien CA, Gubrij I, Lin S-C, Saylors RL, Manolagas SC (1999) STAT3 Activation in Stromal/Osteoblastic Cells Is Required for Induction of the Receptor Activator of NF-κB Ligand and Stimulation of Osteoclastogenesis by gp130-utilizing Cytokines or Interleukin-1 but Not 1,25-Dihydroxyvitamin D3 or Parathyroid Hormone. Journal of Biological Chemistry 274: 19301–19308. 10383440 10.1074/jbc.274.27.19301

[101] Bishop KA, Meyer MB, Pike JW (2009) A Novel Distal Enhancer Mediates Cytokine Induction of Mouse Rankl Gene Expression. Molecular Endocrinology 23: 2095–2110. 19880655 10.1210/me.2009-0209 PMC2796147

[102] Mori T, Miyamoto T, Yoshida H, Asakawa M, Kawasumi M, et al (2011) IL-1b and TNFa-initiated IL-6–STAT3 pathway is critical in mediating inflammatory cytokines and RANKL expression in inflammatory arthritis. International Immunology 23: 701–712. 21937456 10.1093/intimm/dxr077

[103] Zanotti S, Stadmeyer L, Smerdel-Ramoya A, Durant D, Canalis E (2009) Misexpression of CCAAT/enhancer binding protein beta causes osteopenia. Journal of Endocrinology 201: 263–274. 19218285 10.1677/JOE-08-0514 PMC2674520

[104] Ng PK-S, Tsui SK-W, Lau CP-Y, Wong C-H, Wong WH-T, et al (2010) CCAAT/enhancer binding protein beta is up-regulated in giant cell tumor of bone and regulates RANKL expression. Journal of Cellular Biochemistry 110: 438–446. 20225273 10.1002/jcb.22556

[105] Zamurovic N, Cappellen D, Rohner D, Susa M (2004) Coordinated Activation of Notch, Wnt, and Transforming Growth Factor-β Signaling Pathways in Bone Morphogenic Protein 2-induced Osteogenesis. Journal of Biological Chemistry 279: 37704–37715. 15178686 10.1074/jbc.M403813200

[106] Xing Q, Ye Q, Fan M, Zhou Y, Xu Q, et al (2010) Porphyromonas gingivalis lipopolysaccharide inhibits the osteoblastic differentiation of preosteoblasts by activating Notch1 signaling. Journal of Cellular Physiology 225: 106–114. 20648628 10.1002/jcp.22201

[107] Hayden MS, Ghosh S (2012) NF-κB, the first quarter-century: remarkable progress and outstanding questions. Genes & Development 26: 203–234. 22302935 10.1101/gad.183434.111 PMC3278889

[108] Arabaci T, Cicek Y, Canakci V, Canakci CF, Ozgoz M, et al (2010) Immunohistochemical and Stereologic Analysis of NF-κB Activation in Chronic Periodontitis. European Journal of Dentistry 4: 454–461. 20922166 PMC2948733

[109] Sun Z, Andersson R (2002) NF-kB Activation and Inhibition: A Review. Shock 18: 99–106. 12166787 10.1097/00024382-200208000-00001

[110] Choi E-K, Park S-A, Oh W-M, Kang H-C, Kuramitsu HK, et al (2005) Mechanisms of Porphyromonas gingivalis-induced monocyte chemoattractant protein-1 expression in endothelial cells. FEMS Immunology & Medical Microbiology 44: 51–58. 15780578 10.1016/j.femsim.2004.12.003

[111] Walter C, Zahlten J, Schmeck B, Schaudinn C, Hippenstiel S, et al (2004) Porphyromonas gingivalis Strain-Dependent Activation of Human Endothelial Cells. Infection and Immunity 72: 5910–5918. 15385493 10.1128/IAI.72.10.5910-5918.2004 PMC517532

[112] Zhang D, Zheng H, Zhao J, Lin L, Li C, et al (2011) Porphorymonas gingivalis induces intracellular adhesion molecule-1 expression in endothelial cells through the nuclear factor-kappaB pathway, but not through the p38 MAPK pathway. Journal of Periodontal Research 46: 31–38. 20825555 10.1111/j.1600-0765.2010.01305.x

[113] Yanti, Lee M, Kim D, Hwang J-K (2009) Inhibitory Effect of Panduratin A on c-Jun N-Terminal Kinase and Activator Protein-1 Signaling Involved in Porphyromonas gingivalis Supernatant-Stimulated Matrix Metalloproteinase-9 Expression in Human Oral Epidermoid Cells. Biological and Pharmaceutical Bulletin 32: 1770–1775. 19801842 10.1248/bpb.32.1770

[114] Wong ML, Medrano JF (2005) Real-time PCR for mRNA quantitation. Biotechniques 39: 75–85. 16060372 10.2144/05391RV01

