# Supplementary material for: Transcriptional Profiling of Bone Marrow Stromal Cells in Response to Porphyromonas gingivalis Secreted Products
Source: PLoS One. 2012 Aug 24;7(8):e43899. doi: 10.1371/journal.pone.0043899 (PMC3427182; doi:10.1371/journal.pone.0043899)
Supplement: Table S1 — List of genes regulated after 6 h in response to Porphyromonas gingivalis. (DOC) [file pone.0043899.s001.doc]

## Table S1. List of genes regulated after 6 h in response to *Porphyromonas gingivalis*

**A) Genes up-regulated more than two-fold at 6 h**

| **Gene symbol** | **Fold change** |
| --- | --- |
| Saa3 | 144.7943 |
| Lcn2 | 33.58387 |
| Cxcl1 | 23.13512 |
| Ccl2 | 17.62608 |
| Ptx3 | 11.93013 |
| Ptx3 | 11.52533 |
| Nfkbiz | 11.212 |
| Cx3cl1 | 10.57374 |
| Cxcl9 | 10.49606 |
| Mmp13 | 9.319547 |
| Gch1 | 9.005278 |
| Ccl5 | 8.939191 |
| Ch25h | 8.355517 |
| Ccl17 | 7.965845 |
| Mmp13 | 7.810667 |
| Fas | 7.78019 |
| Ccl9 | 7.167534 |
| Cxcl10 | 7.142059 |
| Fas | 6.517889 |
| Serpina3g | 6.313765 |
| Cd74 | 6.176455 |
| Ankrd47 | 5.427477 |
| Cdkn1a | 5.263454 |
| Cd74 | 5.025454 |
| Slpi | 4.940808 |
| Rem2 | 4.937027 |
| Ier3 | 4.916338 |
| Slc2a6 | 4.719902 |
| Nfkbia | 4.609359 |
| Zc3h12a | 4.56093 |
| Gbp2 | 4.374025 |
| Slc24a6 | 4.008769 |
| Tmem25 | 3.955971 |
| TLR-2 | 3.744937 |
| Syngr3 | 3.478439 |
| Mmp17 | 3.405849 |
| Cebpb | 3.373075 |
| Insl6 | 3.370797 |
| Ccl3 | 3.34252 |
| 6430548M08Rik | 3.331163 |
| Hrbl | 3.295758 |
| Chac1 | 3.280644 |
| Plk2 | 3.266554 |
| Ptgs2 | 3.236976 |
| 4930572J05Rik | 3.235112 |
| Ehd3 | 3.222709 |
| Hap1 | 3.221821 |
| Grtp1 | 3.203551 |
| LOC100044177 | 3.197671 |
| Ptges | 3.183894 |
| Ifi47 | 3.119106 |
| Mycl1 | 3.085585 |
| Syn1 | 3.078951 |

| **Gene symbol** | **Fold change** |
| --- | --- |
| Nfkbie | 3.056928 |
| Sc4mol | 3.045707 |
| 1810015C04Rik | 2.994005 |
| LOC100047651 | 2.981218 |
| Sqle | 2.92861 |
| Reep6 | 2.916075 |
| LOC100043986 | 2.910129 |
| Spsb1 | 2.872737 |
| Ttll1 | 2.869438 |
| Csdc2 | 2.855875 |
| H1f0 | 2.843256 |
| Ccl7 | 2.832705 |
| Mapk8ip1 | 2.829642 |
| Dcxr | 2.803246 |
| Rasl11b | 2.801031 |
| Ccrn4l | 2.799202 |
| Pfkp | 2.797755 |
| Egr2 | 2.793851 |
| Hsd17b7 | 2.786798 |
| Stac2 | 2.784077 |
| Hr | 2.772023 |
| Gcnt2 | 2.771199 |
| Casp4 | 2.766445 |
| Ncald | 2.766394 |
| Pbx4 | 2.764175 |
| Nfkbie | 2.73773 |
| Wfdc3 | 2.73354 |
| Ckb | 2.718222 |
| Tnfaip2 | 2.716493 |
| Casp4 | 2.698254 |
| Tnip1 | 2.697194 |
| Csf1 | 2.68803 |
| Lrig1 | 2.687697 |
| Syn1 | 2.687652 |
| Tnip1 | 2.677298 |
| Stx6 | 2.673198 |
| Rasl11b | 2.666019 |
| Stx6 | 2.664597 |
| Plvap | 2.660739 |
| Eno2 | 2.650579 |
| Smox | 2.648488 |
| Map3k8 | 2.633911 |
| Hp | 2.633588 |
| Junb | 2.630128 |
| Smox | 2.625651 |
| Irf1 | 2.623241 |
| Eif4e3 | 2.617205 |
| Sema6b | 2.611151 |
| Stat3 | 2.609693 |
| 9130213B05Rik | 2.609548 |
| 1700088E04Rik | 2.608456 |
| Irf1 | 2.596547 |
| Serpina3f | 2.59493 |

**A) Genes up-regulated more than two-fold at 6 h (continued)**

| **Gene symbol** | **Fold change** |
| --- | --- |
| Cxcl2 | 2.593425 |
| Hp | 2.583361 |
| Egln3 | 2.579902 |
| Tmod1 | 2.564519 |
| Mfsd7 | 2.564324 |
| Dcxr | 2.545471 |
| Slc45a4 | 2.543356 |
| Hook2 | 2.542683 |
| Usp2 | 2.541824 |
| Ccrn4l | 2.540355 |
| Mapk8ip1 | 2.538546 |
| Por | 2.534494 |
| Plscr1 | 2.524827 |
| Irf1 | 2.50094 |
| Prss35 | 2.497494 |
| Plscr1 | 2.497336 |
| C630004H02Rik | 2.490863 |
| 2810003C17Rik | 2.490143 |
| Chst8 | 2.480563 |
| Nab1 | 2.478614 |
| Snapc1 | 2.43859 |
| Tnfaip3 | 2.435512 |
| Lmcd1 | 2.434751 |
| Mapkapk3 | 2.432875 |
| Ttll1 | 2.430195 |
| Slc11a2 | 2.426307 |
| Nab1 | 2.423758 |
| Por | 2.404097 |
| Tgoln1 | 2.402764 |
| Tubb2b | 2.400274 |
| Cmbl | 2.397074 |
| Csf2 | 2.39652 |
| Tmem66 | 2.396075 |
| Ripk2 | 2.390463 |
| Kank3 | 2.38627 |
| Lmcd1 | 2.385232 |
| Acss2 | 2.374224 |
| Cd248 | 2.371193 |
| Add3 | 2.370945 |
| Zc3h12a | 2.36987 |
| Hp | 2.36574 |
| Smox | 2.359704 |
| Stat3 | 2.359676 |
| Ptges | 2.355503 |
| Sod2 | 2.347944 |
| Tubb2b | 2.338117 |
| Lmcd1 | 2.331752 |
| Capn5 | 2.328319 |
| Rab3a | 2.319274 |
| Brp17 | 2.304231 |
| Axud1 | 2.302903 |
| Crip2 | 2.300811 |
| Idi1 | 2.298137 |

| **Gene symbol** | **Fold change** |
| --- | --- |
| Socs3 | 2.297671 |
| Ifi30 | 2.290553 |
| Gnb5 | 2.289578 |
| BC046404 | 2.289485 |
| Rab3d | 2.276528 |
| Gchfr | 2.27334 |
| Id1 | 2.266497 |
| Skil | 2.265962 |
| Gca | 2.265059 |
| Slc9a3r1 | 2.263847 |
| Mtap | 2.261651 |
| Tnfaip2 | 2.255719 |
| Neu1 | 2.249049 |
| Arhgef3 | 2.248939 |
| Tnfaip2 | 2.247883 |
| Ctsl | 2.240973 |
| Cd44 | 2.235742 |
| Fbxo31 | 2.234761 |
| Igsf9 | 2.224286 |
| Cox7a1 | 2.220156 |
| Pdxp | 2.215614 |
| Asl | 2.212557 |
| Ccdc92 | 2.208948 |
| Limk1 | 2.204789 |
| Eif4e3 | 2.201129 |
| Slc25a33 | 2.200031 |
| Arrb2 | 2.198583 |
| Sqle | 2.188847 |
| BC004044 | 2.188441 |
| Tapbp | 2.188329 |
| Dhcr7 | 2.18483 |
| Rbm38 | 2.183763 |
| P4ha2 | 2.180856 |
| Atp6v1a | 2.176066 |
| Smox | 2.173456 |
| Itpka | 2.171723 |
| Agrn | 2.17011 |
| Gch1 | 2.168017 |
| Irf1 | 2.165895 |
| Prkcd | 2.165097 |
| Rbm38 | 2.162089 |
| Micall2 | 2.161063 |
| Rasl12 | 2.160551 |
| LOC100045343 | 2.159308 |
| Smox | 2.155073 |
| Ubd | 2.151097 |
| Efna3 | 2.145273 |
| Hs2st1 | 2.144169 |
| Ptpn1 | 2.142118 |
| 3010026O09Rik | 2.140865 |
| Ifi30 | 2.134852 |
| Pcyt2 | 2.130996 |
| Hif1a | 2.128772 |

**A) Genes up-regulated more than two-fold at 6 h (continued)**

| **Gene symbol** | **Fold change** |
| --- | --- |
| Gprc5a | 2.128504 |
| Ddit4 | 2.128387 |
| St3gal5 | 2.127805 |
| 1200002N14Rik | 2.127507 |
| Fkbp5 | 2.126505 |
| Rac3 | 2.125568 |
| Bckdhb | 2.123221 |
| Aldh4a1 | 2.12188 |
| Pcyt2 | 2.118279 |
| Lrrc15 | 2.117019 |
| Apol9b | 2.115069 |
| Kif5c | 2.112051 |
| Mtap | 2.106985 |
| Gdf15 | 2.106466 |
| Pcyt2 | 2.106435 |
| Gdpd5 | 2.10298 |
| Napa | 2.09413 |
| A330021E22Rik | 2.092574 |
| LOC100046457 | 2.091888 |
| VEGF | 2.091474 |
| Cbara1 | 2.083462 |
| Phlda1 | 2.080142 |
| Tmem50b | 2.079821 |
| Metrn | 2.073238 |
| Mfap3l | 2.073099 |
| Psmb8 | 2.07089 |
| Snx18 | 2.070212 |
| Hey1 | 2.064908 |
| Fbxo31 | 2.064848 |
| Ttll1 | 2.06149 |
| Tubb2b | 2.059472 |
| Tec | 2.059405 |
| Stat3 | 2.059314 |
| VEGF | 2.055166 |
| Cdk2ap2 | 2.050331 |
| Hagh | 2.04793 |
| LOC100048436 | 2.0459 |
| Gnb5 | 2.042187 |
| Rab3il1 | 2.041382 |
| H2-DMa | 2.040253 |
| Tsc22d1 | 2.039001 |
| Cdkn2a | 2.036461 |
| H2-M3 | 2.032903 |
| Hrb | 2.030487 |
| Atp6v0b | 2.027443 |
| Dok4 | 2.024406 |
| Syt13 | 2.020575 |
| Col18a1 | 2.01886 |
| Dos | 2.018582 |
| Rab3ip | 2.017181 |
| Igsf3 | 2.016906 |
| Arntl | 2.01374 |
| Rpo2tc1 | 2.01185 |

| **Gene symbol** | **Fold change** |
| --- | --- |
| Xpr1 | 2.010442 |
| Siat9 | 2.01002 |
| Asl | 2.008372 |
| Plxdc1 | 2.002261 |
| Bex2 | 2.000896 |
| Tnnc1 | 2.000369 |
| Xpr1 | 2.010442 |
| Siat9 | 2.01002 |

**B) Genes down-regulated more than two-fold at 6 h**

| **Gene symbol** | **Fold change** |
| --- | --- |
| Cc2d2a | -4.68022 |
| Sema3a | -4.51999 |
| Foxg1 | -4.19903 |
| Zfp532 | -4.1979 |
| Tnfrsf11b | -4.01238 |
| LOC100044776 | -3.95499 |
| Il1rn | -3.93934 |
| Ppapdc1 | -3.67174 |
| Zfp532 | -3.65987 |
| Dbp | -3.45868 |
| S3-12 | -3.42361 |
| Zfh4 | -3.40096 |
| Pik3ip1 | -3.38379 |
| Dis3l | -3.29722 |
| Rasl11a | -3.23969 |
| Pet112l | -3.23761 |
| Pet112l | -3.2306 |
| Rspo2 | -3.17911 |
| Tgf-b1i1 | -3.16534 |
| 0610037L13Rik | -3.16189 |
| Mylk | -3.14287 |
| LOC100046883 | -3.11348 |
| Tgf-b1i1 | -3.07427 |
| 1700034H14Rik | -3.06422 |
| Mylk | -3.06158 |
| Trp63 | -3.03827 |
| Cdc20 | -2.9623 |
| Htra3 | -2.91864 |
| Cldn15 | -2.91821 |
| AW061290 | -2.90252 |
| Avpr1a | -2.86738 |
| Il1rn | -2.86103 |
| Zfp521 | -2.85519 |
| Cdc20 | -2.84092 |
| Suv420h1 | -2.81633 |
| LOC100046044 | -2.76721 |
| Fbxo4 | -2.7586 |
| Hcfc1 | -2.68577 |
| Tbc1d5 | -2.67593 |
| 1700030K09Rik | -2.67589 |
| Brd9 | -2.6697 |
| 1700034H14Rik | -2.65959 |
| N4bp2 | -2.65808 |
| A930025D01Rik | -2.65799 |
| Fes | -2.65303 |
| Rpe | -2.64386 |
| 4833442J19Rik | -2.6403 |
| Wisp2 | -2.6395 |
| Cul7 | -2.63742 |
| Gtf2ird2 | -2.62073 |
| Mfap5 | -2.61579 |
| Setd1b | -2.59592 |
| Gper | -2.5917 |

| **Gene symbol** | **Fold change** |
| --- | --- |
| Kctd12b | -2.58695 |
| Ap4s1 | -2.55583 |
| 1700034H14Rik | -2.52236 |
| D14Ertd449e | -2.52112 |
| Fbxo6 | -2.51656 |
| Efna5 | -2.51226 |
| Aven | -2.49702 |
| Myl4 | -2.49125 |
| N6amt1 | -2.48415 |
| Figf | -2.47872 |
| Tnfrsf12a | -2.47506 |
| Epc1 | -2.47061 |
| 1500012F01Rik | -2.46481 |
| Pet112l | -2.46465 |
| Ap4m1 | -2.45563 |
| Pank1 | -2.44759 |
| A930025D01Rik | -2.44651 |
| Tor1aip1 | -2.4448 |
| Runx1t1 | -2.43824 |
| Lgr5 | -2.43648 |
| Prrg2 | -2.42328 |
| BC067068 | -2.41687 |
| Ela1 | -2.40697 |
| Irx5 | -2.39742 |
| Adam12 | -2.39657 |
| Mcph1 | -2.39006 |
| Gpr176 | -2.38935 |
| Pet112l | -2.38924 |
| Ulk2 | -2.38357 |
| Epc1 | -2.38284 |
| Trub1 | -2.37871 |
| Taf9b | -2.37527 |
| Tmco4 | -2.37516 |
| 2410016F19Rik | -2.37425 |
| Pus3 | -2.37366 |
| Smo | -2.36099 |
| Trub1 | -2.35949 |
| Accs | -2.35649 |
| Npr3 | -2.35556 |
| Tceal8 | -2.35316 |
| Mtrf1 | -2.35131 |
| Nr1h3 | -2.34465 |
| Rpap1 | -2.32809 |
| Nr1d2 | -2.3242 |
| Md-2 | -2.32219 |
| Snx33 | -2.31425 |
| Evi2a | -2.30309 |
| Brd9 | -2.30293 |
| Pkig | -2.30141 |
| Suv420h1 | -2.29587 |
| Mc5r | -2.29446 |
| D030070L09Rik | -2.29126 |
| Jmjd5 | -2.29075 |

Genes downregulated more than two-fold at 6 h continued

| **Gene symbol** | **Fold change** |
| --- | --- |
| Traf3ip2 | -2.28773 |
| Gulp1 | -2.28493 |
| 6720458F09Rik | -2.28161 |
| Ccdc91 | -2.28075 |
| Gtf2ird2 | -2.27295 |
| Lrrk1 | -2.2724 |
| 1110001A07Rik | -2.26965 |
| Sspn | -2.26937 |
| Ndufaf1 | -2.26834 |
| Gtf3c3 | -2.26806 |
| Nr2f2 | -2.25612 |
| Cul1 | -2.24492 |
| Txnl4b | -2.24427 |
| Slc39a10 | -2.23987 |
| Scmh1 | -2.23846 |
| 1700025K23Rik | -2.23689 |
| Bmp4 | -2.23541 |
| Tsga14 | -2.23052 |
| Plk1 | -2.22812 |
| Rcbtb2 | -2.22794 |
| Elf1 | -2.22759 |
| Mcrs1 | -2.22164 |
| Tmem42 | -2.22163 |
| 2010321M09Rik | -2.22041 |
| Qrsl1 | -2.2171 |
| Hirip3 | -2.20834 |
| Arsj | -2.20612 |
| Nrn1 | -2.20592 |
| Acsf3 | -2.20041 |
| Tpk1 | -2.19297 |
| Spag7 | -2.18816 |
| Stag1 | -2.18815 |
| Epdr1 | -2.18706 |
| Spon2 | -2.18274 |
| D030070L09Rik | -2.17048 |
| Mmp14 | -2.16416 |
| Pik3c3 | -2.16369 |
| Avpr1a | -2.16249 |
| C87436 | -2.16006 |
| Pcdhb22 | -2.15989 |
| Thnsl2 | -2.15669 |
| Il1rl1l | -2.15075 |
| Nsmaf | -2.14731 |
| Tmed1 | -2.14439 |
| Zfp236 | -2.13389 |
| Tarbp2 | -2.13156 |
| Gpx7 | -2.12866 |
| Fntb | -2.12605 |
| Aurka | -2.12591 |
| 6530404N21Rik | -2.12043 |
| 2610524A10Rik | -2.11754 |
| 1500010J02Rik | -2.11563 |
| Gbl | -2.10685 |

| **Gene symbol** | **Fold change** |
| --- | --- |
| Thtpa | -2.10438 |
| Rnf103 | -2.10268 |
| Nt5m | -2.0996 |
| Ing4 | -2.09917 |
| Gtrgeo22 | -2.09713 |
| Krt10 | -2.09708 |
| Hbp1 | -2.09662 |
| 1810009N02Rik | -2.09599 |
| Gnpda2 | -2.08813 |
| Rhobtb3 | -2.08728 |
| Tead2 | -2.07968 |
| Tgf-b3 | -2.07954 |
| Lrba | -2.07926 |
| Sdpr | -2.07774 |
| 2010321M09Rik | -2.07661 |
| Ubtf | -2.07384 |
| Ppa2 | -2.07209 |
| Tapbpl | -2.07173 |
| Tmem17 | -2.07136 |
| Mbd3 | -2.0694 |
| Htra3 | -2.06737 |
| Zfp710 | -2.06636 |
| Rab11b | -2.0636 |
| Hsd3b7 | -2.06044 |
| BC029169 | -2.05888 |
| Hmgb2 | -2.05529 |
| Frk | -2.0525 |
| 2610002M06Rik | -2.05154 |
| Npepl1 | -2.04865 |
| 2700062C07Rik | -2.04637 |
| Zfp236 | -2.04508 |
| Tmem209 | -2.04433 |
| Acy3 | -2.0413 |
| Mrpl48 | -2.04005 |
| BC039093 | -2.03696 |
| Eif5 | -2.03329 |
| Acp2 | -2.03292 |
| Cdkn2c | -2.02853 |
| Gbl | -2.02591 |
| Pbk | -2.02301 |
| Kcnk2 | -2.02158 |
| Gtf2h4 | -2.02099 |
| Nusap1 | -2.02019 |
| 5430435G22Rik | -2.02017 |
| Cenpa | -2.01976 |
| Pdgfra | -2.0154 |
| C87436 | -2.01401 |
| 2610044O15Rik | -2.01325 |
| Prkcbp1 | -2.00829 |
| N6amt1 | -2.00617 |
| Thtpa | -2.10438 |
| Rnf103 | -2.10268 |
| Nt5m | -2.0996 |
