# Supplementary material for: Transcriptional Profiling of Bone Marrow Stromal Cells in Response to Porphyromonas gingivalis Secreted Products
Source: PLoS One. 2012 Aug 24;7(8):e43899. doi: 10.1371/journal.pone.0043899 (PMC3427182; doi:10.1371/journal.pone.0043899)
Supplement: Table S2 — List of genes regulated after 24 h in response to Porphyromonas gingivalis. (DOC) [file pone.0043899.s002.doc]

## Table S2.List of genes regulated after 24 h in response to *Porphyromonas gingivalis*

**A) Genes up-regulated more than two-fold at 24 h**

| **Gene symbol** | **Fold change** |
| --- | --- |
| Lcn2 | 354.7951 |
| Saa3 | 143.8862 |
| LOC100044702 | 111.9489 |
| Ccl5 | 86.75045 |
| Mmp3 | 35.80779 |
| Cxcl9 | 34.95269 |
| Ptx3 | 31.98103 |
| Cxcl1 | 29.22046 |
| Ptx3 | 27.63012 |
| Ch25h | 24.79245 |
| Il-6 | 23.2266 |
| Ccl2 | 20.68476 |
| Cfb | 19.94681 |
| Slpi | 19.45854 |
| Mmp3 | 16.91814 |
| Gbp2 | 16.32286 |
| Ppbp | 15.96733 |
| Ppbp | 14.37349 |
| C3 | 14.32161 |
| Cd74 | 13.14656 |
| TLR-2 | 12.6473 |
| Fas | 11.48997 |
| Il13ra2 | 10.90555 |
| Cbr2 | 10.71476 |
| Gbp3 | 10.23904 |
| Cd74 | 9.774575 |
| Nfkbiz | 9.56099 |
| Camp | 9.350696 |
| Mmp3 | 9.128228 |
| Fas | 8.878448 |
| Serpinb9g | 8.687324 |
| Mmp13 | 8.439232 |
| Ccl9 | 8.268634 |
| Plk2 | 7.582802 |
| Hp | 7.483097 |
| Zc3h12a | 7.326866 |
| Mmp13 | 7.29968 |
| Hpx | 7.023619 |
| Hp | 7.001449 |
| Slc2a6 | 7.001397 |
| Serpina3g | 6.977099 |
| Jak2 | 6.904497 |
| Rac3 | 6.876009 |
| Cxcl15 | 6.772895 |
| Mt2 | 6.511619 |
| IL-1α | 6.450745 |
| Slc15a3 | 6.305687 |
| Gbp3 | 6.190493 |
| Dnmt3l | 6.156311 |
| Osmr | 6.077178 |
| Rac3 | 6.024558 |
| Tnip1 | 5.939297 |
| Cxcl10 | 5.90929 |
| 1100001G20Rik | 5.837463 |
| Usp18 | 5.75763 |
| Nfkbia | 5.736463 |

| **Gene symbol** | **Fold change** |
| --- | --- |
| LOC435337 | 5.705378 |
| Serpina3f | 5.664974 |
| Oasl2 | 5.629959 |
| Dnmt3l | 5.62634 |
| Nfkbie | 5.53638 |
| Jak2 | 5.533565 |
| LOC100048346 | 5.50133 |
| Ier3 | 5.477968 |
| Vnn3 | 5.447197 |
| Junb | 5.356735 |
| Ly6c1 | 4.924612 |
| Tnfaip3 | 4.751849 |
| Cx3cl1 | 4.690369 |
| Tnip1 | 4.524131 |
| LOC100038882 | 4.50169 |
| Socs3 | 4.454419 |
| Hp | 4.341765 |
| Vnn3 | 4.326532 |
| Casp4 | 4.313249 |
| Il13ra1 | 4.126325 |
| 1200002N14Rik | 4.182948 |
| Cebpb | 4.180982 |
| Zc3h12a | 4.167035 |
| Map3k8 | 4.150677 |
| RELB | 3.998608 |
| Dcxr | 3.978684 |
| Casp4 | 3.941854 |
| Tnfrsf9 | 3.874082 |
| Bst2 | 3.779415 |
| Mfsd7 | 3.752104 |
| Slc7a2 | 3.699996 |
| Ccl7 | 3.693088 |
| Gbp2 | 3.676518 |
| Rcan1 | 3.586354 |
| Usp18 | 3.521841 |
| Rcl1 | 3.51005 |
| Nfkbie | 3.484758 |
| Angptl4 | 3.465131 |
| Dcxr | 3.454607 |
| H2-Ab1 | 3.43524 |
| Tnfsf13b | 3.387728 |
| S100a8 | 3.381834 |
| Ereg | 3.370088 |
| Actc1 | 3.352229 |
| Abcc3 | 3.27527 |
| Ubd | 3.230891 |
| Sod2 | 3.22047 |
| Iigp2 | 3.201707 |
| Wfdc12 | 3.183889 |
| Ifitm3 | 3.178828 |
| Gja8 | 3.178367 |
| Tnfsf13b | 3.169215 |
| 4933426M11Rik | 3.147578 |
| AU018778 | 3.142585 |

**A) Genes up-regulated more than two-fold at 24 h (continued)**

| **Gene symbol** | **Fold change** |
| --- | --- |
| Ccl17 | 3.136138 |
| 4930583H14Rik | 3.120409 |
| LOC100048710 | 3.117704 |
| Serping1 | 3.113135 |
| Ppp1r15b | 3.073311 |
| Cxcl2 | 3.024402 |
| Ifi27 | 2.988571 |
| Slc7a2 | 2.981963 |
| Trib3 | 2.97513 |
| Rcl1 | 2.961909 |
| Mmp9 | 2.95792 |
| Enpp2 | 2.938036 |
| Ripk2 | 2.914385 |
| Matn4 | 2.91226 |
| Ifi47 | 2.909141 |
| Mmp12 | 2.89754 |
| Dgat2 | 2.893499 |
| Igtp | 2.854418 |
| Cox7a1 | 2.826698 |
| Rcl1 | 2.814322 |
| Riok3 | 2.792285 |
| Egfl7 | 2.787763 |
| Plscr1 | 2.783824 |
| H2-T23 | 2.783414 |
| Orm2 | 2.76792 |
| Csgalnact1 | 2.758847 |
| Ube2l6 | 2.756154 |
| VEGF | 2.752308 |
| H2-Q7 | 2.740188 |
| Hif1a | 2.74015 |
| Ly6a | 2.733837 |
| Dcn | 2.722417 |
| EG630499 | 2.715031 |
| Casp4 | 2.714532 |
| Insl6 | 2.698915 |
| Arhgdib | 2.696723 |
| Dhx58 | 2.690421 |
| H2-Ab1 | 2.684702 |
| Ets2 | 2.677864 |
| Dcn | 2.67034 |
| Clip1 | 2.663725 |
| Bcl3 | 2.655449 |
| Prrx1 | 2.654502 |
| Ifnar2 | 2.653588 |
| Tmem176b | 2.640099 |
| Chac1 | 2.639009 |
| LOC547343 | 2.638172 |
| Stat3 | 2.622056 |
| D14Ertd668e | 2.603786 |
| Clip1 | 2.601327 |
| Hapln4 | 2.598229 |
| A4galt | 2.598076 |
| C1s | 2.596423 |

| **Gene symbol** | **Fold change** |
| --- | --- |
| Rsn | 2.591058 |
| LOC100046232 | 2.586748 |
| Nfkb1 | 2.567591 |
| Plscr1 | 2.565908 |
| Gadd45b | 2.564189 |
| D12Ertd647e | 2.549963 |
| Ifnar2 | 2.547994 |
| Ifnar2 | 2.542844 |
| Snx18 | 2.531356 |
| Gp38 | 2.522789 |
| Slco1a5 | 2.516495 |
| P2rx4 | 2.514034 |
| Tnip1 | 2.512111 |
| C1s | 2.509864 |
| LOC626578 | 2.508939 |
| 9130213B05Rik | 2.50837 |
| P2rx4 | 2.500625 |
| Gdnf | 2.49757 |
| VEGF | 2.480708 |
| Plscr2 | 2.464677 |
| Irf1 | 2.463881 |
| Parp14 | 2.447776 |
| Skil | 2.439202 |
| Prrx1 | 2.436542 |
| Gstt1 | 2.431473 |
| Lbp | 2.41984 |
| Stat3 | 2.411036 |
| Irf9 | 2.404343 |
| Susd2 | 2.395802 |
| Adora2b | 2.385239 |
| D12Ertd647e | 2.383773 |
| Slc11a2 | 2.365842 |
| Stat3 | 2.359274 |
| Irf1 | 2.344791 |
| Ctps | 2.33914 |
| Ifit3 | 2.336982 |
| Irf1 | 2.331641 |
| F13a1 | 2.329826 |
| H2-T23 | 2.325076 |
| Riok3 | 2.324276 |
| Hgf | 2.321185 |
| LOC667370 | 2.308213 |
| Irf1 | 2.306419 |
| Npy1r | 2.305056 |
| Cxcl4 | 2.30002 |
| Ddt | 2.300006 |
| Tnfrsf9 | 2.285822 |
| Irak3 | 2.283404 |
| Rasl12 | 2.28032 |
| C4b | 2.276476 |
| Ptgs2 | 2.276068 |
| Prrx1 | 2.269099 |
| Macrod1 | 2.264048 |

**A) Genes up-regulated more than two-fold at 24 h continued**

| **Gene symbol** | **Fold change** |
| --- | --- |
| Serpinb6b | 2.263698 |
| Gypc | 2.262248 |
| Lrrc8 | 2.246792 |
| Mmp13 | 2.245348 |
| Col18a1 | 2.234789 |
| Ddx58 | 2.209375 |
| Trim47 | 2.20855 |
| Cdc42ep3 | 2.201048 |
| Sars | 2.199899 |
| Asns | 2.189598 |
| Ctps | 2.179705 |
| Nfkb2 | 2.173142 |
| Sbno2 | 2.165198 |
| LOC56628 | 2.163463 |
| Nadk | 2.149603 |
| Tmod1 | 2.1477 |
| Rrbp1 | 2.134948 |
| Ctps | 2.12349 |
| Cd14 | 2.121116 |
| D14Ertd668e | 2.120823 |
| Ifit3 | 2.119507 |
| Akp2 | 2.115837 |
| Sphk1 | 2.103179 |
| Asns | 2.100091 |
| Pla1a | 2.097374 |
| Rffl | 2.097132 |
| Psmd10 | 2.088839 |
| Tcp10a | 2.084519 |
| Timp1 | 2.07527 |
| LOC100044190 | 2.067314 |
| Slit2 | 2.061944 |
| Adamts7 | 2.055568 |
| Dusp16 | 2.052084 |
| Rhou | 2.049477 |
| Il10rb | 2.048616 |
| A630077B13Rik | 2.041595 |
| EG317677 | 2.040942 |
| H2-M3 | 2.035108 |
| Bckdhb | 2.032184 |
| 1110008P14Rik | 2.030749 |
| Gstt3 | 2.030136 |
| Cyp4f14 | 2.025628 |
| Dtwd1 | 2.018756 |
| Nab1 | 2.015766 |
| Lmo4 | 2.012415 |
| 2010002N04Rik | 2.009882 |
| CD68 | 2.001726 |
| Lxn | 2.00117 |

**B) Genes down-regulated more than two-fold at 24 h**

| **Gene symbol** | **Fold change** |
| --- | --- |
| Sult1a1 | -4.62267 |
| Krt13 | -4.41691 |
| Gper | -4.38766 |
| Aldh3a1 | -3.41507 |
| Osr2 | -3.31904 |
| Cldn15 | -3.19474 |
| Slc40a1 | -3.163 |
| Inmt | -3.13242 |
| Ccnd1 | -3.12871 |
| Sox9 | -3.06391 |
| Il17rd | -3.01461 |
| Efnb2 | -2.99538 |
| Ctnnal1 | -2.95605 |
| Ptprv | -2.94636 |
| Ermp1 | -2.93965 |
| Mgp | -2.92179 |
| Spon2 | -2.84905 |
| Htra3 | -2.82317 |
| Ppl | -2.75327 |
| Fdxr | -2.7242 |
| Sep-06 | -2.66337 |
| Efnb1 | -2.64868 |
| Abca9 | -2.60776 |
| Ppp1r3c | -2.59233 |
| Lbh | -2.56834 |
| Ccnd1 | -2.56352 |
| Cldn15 | -2.5557 |
| Pdk4 | -2.52822 |
| Hist1h2ah | -2.49804 |
| Hist1h2an | -2.47889 |
| Tgf-b3 | -2.47749 |
| Fdxr | -2.46582 |
| Ndrg1 | -2.46309 |
| Rin2 | -2.44748 |
| Tgm5 | -2.44181 |
| Sep-06 | -2.43941 |
| Hist1h2af | -2.41087 |
| Pacs2 | -2.39414 |
| Nxph2 | -2.39089 |
| Hist1h2ak | -2.38737 |
| Rarb | -2.38412 |
| Ccnd1 | -2.38366 |
| Cldn15 | -2.38091 |
| Aqp1 | -2.37443 |
| Emp1 | -2.36062 |
| Centg2 | -2.34688 |
| Hist1h2ad | -2.34156 |
| Pxmp4 | -2.33454 |
| Il15 | -2.33392 |
| Rin2 | -2.33246 |
| Il15 | -2.33114 |
| Hist1h2ao | -2.32076 |
| Pdlim2 | -2.31868 |

| **Gene symbol** | **Fold change** |
| --- | --- |
| Evi2a | -2.31441 |
| Cdkn2c | -2.30324 |
| Sertad4 | -2.28371 |
| Prkcdbp | -2.28279 |
| Prr7 | -2.27869 |
| Arntl | -2.26196 |
| Zfp608 | -2.25554 |
| Tgm5 | -2.22698 |
| Kctd12b | -2.21893 |
| Sesn1 | -2.20147 |
| Chst1 | -2.18637 |
| Mmd | -2.18635 |
| Cirbp | -2.18202 |
| Pdlim2 | -2.1765 |
| Hist1h2ag | -2.17517 |
| Mybl2 | -2.17504 |
| Tcf19 | -2.15808 |
| Itga11 | -2.15242 |
| Sema5a | -2.15118 |
| Plxna2 | -2.14688 |
| Nrp1 | -2.14356 |
| Impa2 | -2.13525 |
| Birc5 | -2.13391 |
| Ela1 | -2.12755 |
| Dkk3 | -2.1223 |
| Dkk3 | -2.11273 |
| Cav1 | -2.10508 |
| Mcm5 | -2.09262 |
| Nrp1 | -2.09194 |
| Bok | -2.09175 |
| Birc5 | -2.09138 |
| Vim | -2.07911 |
| LOC100047651 | -2.07516 |
| Dio3 | -2.07201 |
| Drctnnb1a | -2.06527 |
| Gulp1 | -2.05275 |
| Gamt | -2.0469 |
| Vgll4 | -2.04157 |
| Prkcdbp | -2.04101 |
| Bach2 | -2.03487 |
| Pdlim2 | -2.03319 |
| Birc5 | -2.03285 |
| Trp53inp1 | -2.02725 |
| Cdca3 | -2.02658 |
| Hist2h2ab | -2.01658 |
| Zfp521 | -2.01573 |
| Sema5a | -2.01504 |
| Rdm1 | -2.01135 |
| 5430435G22Rik | -2.00689 |
| Ndrl | -2.00502 |
| Wnt5a | -2.00502 |
